# Neuromuscular Diseases Due to Chaperone Mutations: A Review and Some New Results

**DOI:** 10.3390/ijms21041409

**Published:** 2020-02-19

**Authors:** Jaakko Sarparanta, Per Harald Jonson, Sabita Kawan, Bjarne Udd

**Affiliations:** 1 Folkhälsan Research Center, Helsinki, Finland and Medicum, University of Helsinki, FI-00290 Helsinki, Finland; per-harald.jonson@helsinki.fi (P.H.J.); sabita.kawan@helsinki.fi (S.K.); bjarne.udd@netikka.fi (B.U.); 2 Neuromuscular Research Unit, Department of Neurology, University Hospital and University of Tampere, FI-33520 Tampere, Finland; 3 Department of Neurology, Vaasa Central Hospital, FI-65100 Vaasa, Finland

**Keywords:** heat shock protein, J-domain protein, neuropathy, myopathy, pathomechanism

## Abstract

Skeletal muscle and the nervous system depend on efficient protein quality control, and they express chaperones and cochaperones at high levels to maintain protein homeostasis. Mutations in many of these proteins cause neuromuscular diseases, myopathies, and hereditary motor and sensorimotor neuropathies. In this review, we cover mutations in DNAJB6, DNAJB2, αB-crystallin (CRYAB, HSPB5), HSPB1, HSPB3, HSPB8, and BAG3, and discuss the molecular mechanisms by which they cause neuromuscular disease. In addition, previously unpublished results are presented, showing downstream effects of BAG3 p.P209L on DNAJB6 turnover and localization.

## 1. Introduction

Maintaining protein homeostasis is essential for cellular functioning. This is demonstrated by the diversity of the molecular machinery evolved to maintain the protein homeostasis and by the pathologies associated with dysfunctional protein quality control (PQC).

Chaperones, together with their essential cofactors known as cochaperones, assist their client proteins in attaining their native conformation, prevent unfolded or misfolded proteins from aggregation, and target damaged or superfluous proteins to degradative pathways [1]. Efficient PQC requires the interplay of the different chaperone systems [2,3]. Some of these, e.g., the HSPA (Hsp70) family, consume ATP for client-binding cycles, whereas others, such as the small heat shock proteins (HSPB), are energy-independent [1,4]. Cochaperones such as J-domain proteins (JDP, Hsp40) and BAG proteins assist chaperones in their functions, mediate interactions of the different chaperone families, and affect the fate of the client proteins [1,2].

The chaperone systems are tightly connected to the protein turnover pathways, the ubiquitin–proteasome system (UPS), and the autophagy–lysosome system. In UPS, the principal turnover pathway for soluble proteins, target proteins are tagged with polyubiquitin chains and delivered to proteasomes for degradation; both of these steps are mediated by chaperones and cochaperones [5]. The three main autophagic pathways—macroautophagy, chaperone-mediated autophagy (CMA), and (endosomal) microautophagy—all involving chaperones, use lysosomes for the degradation of their cargoes [1,6]. In macroautophagy, the cargo is sequestered by autophagosomes, which subsequently fuse with lysosomes to deliver their contents. In addition to soluble proteins, this pathway can degrade more complex cargo such as protein aggregates or organelles [6]. In CMA and microautophagy, client proteins are delivered directly to lysosomes or endosomes [6].

**Figure 1:**
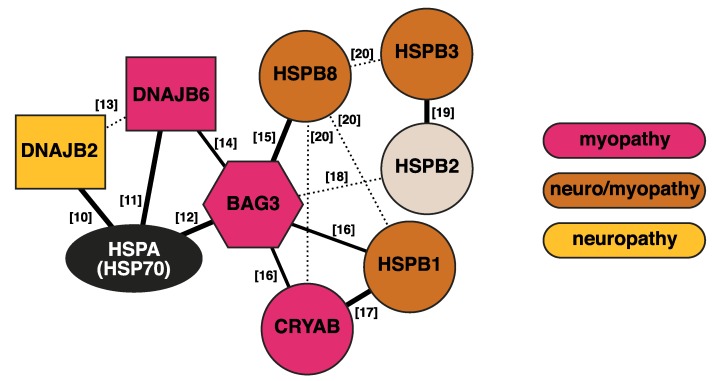
Interaction network of the discussed chaperones and cochaperones. Thick solid lines indicate interactions with established functional relevance. Dashed lines show detected interactions of unknown significance. The numbers indicate references [10,11,12,13,14,15,16,17,18,19,20].

The neuromuscular system, which is responsible for our movements, is largely comprised of post-mitotic, terminally differentiated cells, namely neurons and muscle fibers. These cells have to stay functional through the lifetime of the organism and hence rely on efficient PQC. In muscles, additional challenges are posed by the crowded environment and mechanical, oxidative, and thermal stress, which necessitate the expression of chaperones at high levels [7,8]. Similarly, the long axons of motor neurons are notorious for their susceptibility to damage. Hence, it is not surprising that mutations affecting the PQC system can lead to neuromuscular disease. Indeed, the current version of the gene table of neuromuscular disorders [9] contains at least 15 chaperones or cochaperones (Table 1)—although what can be counted as a chaperone is to some extent a matter of definition. In this review, we will focus on the intimately interconnected network of chaperones and cochaperones presented in Figure 1 and the surprising diversity of pathomechanims by which mutations affecting these proteins cause neuromuscular disease.

## 2. J-Domain Proteins

The J-domain proteins (JDPs), also known as the J-protein or the Hsp40 family, are cochaperones of the ubiquitous HSPA (Hsp70) chaperones [21,22]. The human genome encodes 50 members of the family [23], and these are traditionally divided to class I (DNAJA), class II (DNAJB), and class III (DNAJC) based on their domain structure [22]. The defining feature of JDPs is the J domain (JD), which interacts with the HSPA chaperones through the conserved His–Pro–Asp (HPD) motif [22].

The J domain mediates the canonical function of JDPs—the stimulation of HSPA chaperone activity. HSPAs interact with their clients with alternating low-affinity (ATP-bound) and high-affinity (ADP-bound) states [21]. This HSPA cycle requires JDPs and nucleotide exchange factors (NEFs) as essential cofactors [21]: JDPs stimulate the otherwise very low ATPase activity of HSPAs, thereby promoting the high-affinity client binding. After ATP hydrolysis, NEFs are needed to stimulate the exchange of ADP to ATP and client release [21].

In the DNAJA and DNAJB classes, the N-terminal J domain is followed by a glycine/phenylalanine-rich (G/F) region, which may play different functional roles in different JDPs [22,24]. Based on data from diverse family members, the G/F region may modulate HSPA client binding [25], participate in some client interactions [26], and regulate the HSPA chaperone cycle [27,28].

JDPs of the DNAJA and DNAJB classes differ in the organization of C-terminal parts, which contain the principal client-binding domains [21,29]. DNAJAs harbor a double β barrel domain with a zinc-finger motif, whereas DNAJBs lack the zinc finger and show more variability in the C-terminal domain structure [21,29]. Some members of both classes also contain a C-terminal dimerization domain [21,29]. The DNAJC class—a trash bin for all the JDPs lacking the G/F region and actually the largest class—is structurally and functionally divergent and includes some JDPs with highly specialized functions [21,22,29].

Besides stimulating HSPA ATPase activity, most JDPs themselves recognize and bind non-native proteins; then, they present them to HSPAs [21,29]. In this regard, the diversity of JDPs is thought to provide the HSPA machinery with spatial and functional specificity [21,22]. For example, JDPs do play a role in the triage “decisions” between unfolding and different degradation pathways [1,21].

Similar to the small heat shock proteins discussed below, individual JDPs may have the ability to utilize different binding and action modes, some of which can be HSPA-independent, allowing them to efficiently deal with different types of clients [21,30,31]. The client-binding repertoire of JDPs is further expanded by the recently discovered interclass dimerization between class I and II JDPs, which is utilized in HSPA-mediated protein disaggregation [32,33].

The gene table of neuromuscular disorders currently lists three JDPs as disease genes (Table 1). Mutations in DNAJB6 and DNAJB2 cause myopathy and sensorimotor neuropathy, respectively. These cochaperones, belonging to a subfamily of DNAJBs highly efficient in suppressing protein aggregation [30], will be discussed in more detail below. Spastic ataxia of the Charlevoix–Saguenay type results from recessive mutations in sacsin (*SACS* a.k.a. DNAJC29), which is a large JDP with chaperone and cochaperone activities [34,35,36].

New JDPs may soon be joining the above-mentioned proteins in the neuromuscular gene table. *DNAJB5* was recently identified as a candidate gene for hereditary myoclonus and progressive distal muscular atrophy [37], but its pathogenic role awaits confirmation. DNAJC7, on the other hand, is emerging as a candidate gene for amyotrophic lateral sclerosis (ALS) [38].

### 2.1. DNAJB6

**Figure 2:**
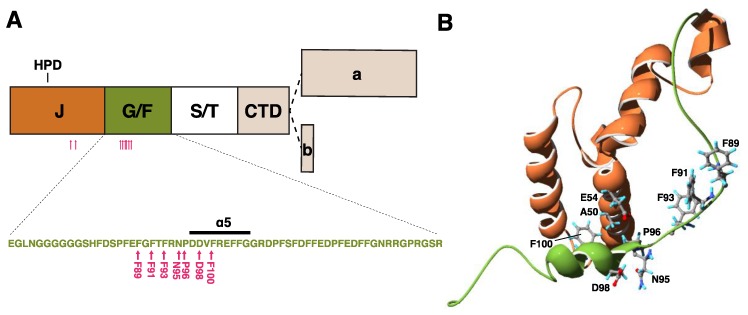
Structure of DNAJB6 and mutations. (**A**) A schematic view of the DNAJB6 protein, with the various domains, and the alternatively spliced C-terminal parts of the “a” and “b” isoforms indicated. The inset shows the sequence of the glycine/phenylalanine-rich (G/F) domain, with the α5 helix and myopathy-causing mutations (pink arrows). (**B**) Protein structure of the J (orange) and G/F (green) domains, with residues harboring disease mutations shown. Structure from Protein Data Bank ID 6U3R [28].

The JDP cochaperone DNAJB6, previously known as MRJ or “mammalian relative of DnaJ” [39], exists as two alternatively spliced isoforms differing in their C-terminal parts—DNAJB6a or DNAJB6(L) (326 aa, 36 kDa) and DNAJB6b or DNAJB6(S) (241 aa, 27 kDa) (Figure 2) [40,41]. The part of the protein shared by both isoforms harbors the N-terminal J domain, the G/F region containing most of disease mutations (see below), and a serine/threonine-rich (S/T) region mediating interactions with client proteins [30,42].

The short isoform DNAJB6b exhibits both cytosolic and nuclear localization, and it has been shown to accumulate to nuclei upon heat shock and hypoxia [11,40,43,44,45,46]. It exists as polydisperse oligomers comprising tens of subunits [28,30,47,48]. The long isoform DNAJB6a contains a nuclear localization signal in its unique C-terminal domain, and it was for long considered exclusively intranuclear [40,42]. However, recently, its localization to the nuclear envelope and the endoplasmic reticulum (ER) was discovered [41].

DNAJB6 is widely expressed; it is present at variable levels in most if not all human and murine tissues [11,39,41]. DNAJB6b shows highest expression in the central nervous system (CNS) and seems to be the predominant isoform in most tissues [11,39,41]. In both human and murine heart, DNAJB6a was reported to be the major isoform and expressed on a high level [41]. Data regarding skeletal muscle are variable: while the Western blot results of Ding et al. indicated a clear predominance of DNAJB6a in human and murine muscles [41], those of Bengoechea et al. showed an isoform ratio of approximately 1:1 in human samples [49]. In any case, the overall expression level of DNAJB6 in skeletal muscle is rather low, which is interesting considering the role of DNAJB6 in myopathy [11,41].

#### 2.1.1. Structure of DNAJB6b

Although several 3D structures of J domains from different JDPs have been solved, no structural information for DNAJB6 was available until recently. In 2018, Söderberg et al. published molecular models of monomeric, dimeric, and oligomeric DNAJB6b based on information obtained from crosslinking, small-angle X-ray scattering, and electron microscopy (EM) experiments [48]. The dimer model featured a client-binding groove formed by the S/T-rich regions of the two monomers [48].

Very recently, a solution structure for DNAJB6b was solved by Karamanos and colleagues who used NMR to study full-length DNAJB6b and a ∆ST-DNAJB6b construct lacking the S/T-rich region, revealing important aspects of the structure–function relationships of DNAJB6 [28].

First, while the G/F region is highly flexible, a part of it forms a stable helix (α5) that interacts with the J domain, regulating its accessibility to HSPA [28]. This helix contains an aspartate–isoleucine/valine–phenylalanine (DI/VF or DIF) motif, mutations in which were previously shown to confer toxicity to *E. coli* DnaJ [27,28].

Second, DNAJB6b oligomers form through the C-terminal part of the C-terminal domain (CTD) and not the S/T-rich region as previously thought [28,30]. The dramatic shift of the equilibrium toward monomers seen with deletion of the S/T-rich region [28,30] was suggested to reflect a role for this region in oligomer nucleation [28]. The deletion of the 10 C-terminal amino acid residues, specific to the DNAJB6b isoform, totally abolished oligomerization [28]. An interesting implication is that the oligomeric organization of DNAJB6a could be radically different. It is of note that while dimerization of DNAJB6b has been suggested [48,50], the NMR structure did not provide further evidence for this [28].

Third, DNAJB6b alternates between open and closed conformations due to transient JD–CTD interactions [28], which were also observed in cross-linking experiments [48]. Based on their findings, Karamanos et al. proposed a DNAJ–HSPA cycle model where the autoinhibitory interaction of the α5 helix to JD is released upon client binding, allowing the binding of HSPA to the JD. After ATP hydrolysis, the α5 helix displaces HSPA, releasing it from DNAJ [28].

#### 2.1.2. Functions of DNAJB6

Although functional studies have concentrated on DNAJB6b, both DNAJB6 isoforms have been implicated in a wide range of cellular functions. We will here focus on the ones relevant for neuromuscular disease.

##### Cochaperone Function

DNAJB6b has been shown to bind and stimulate the constitutively expressed family member HSPA8 (Hsc70, Hsp73) [11,43] and physically interact with HSPA6 [51], but its role(s) as a cochaperone are still incompletely understood. The failure of DNAJB6b to support the recovery of luciferase activity after heat shock suggests that it supports degradation rather than refolding [51].

The association of DNAJB6 with BAG3 and HSPB8 [2,14] links it to the chaperone-assisted selective autophagy (CASA, which is discussed in detail below) [14], but its possible role in this degradative pathway remains uncharacterized. Interactions with other BAG proteins in addition to BAG3 [2] are compatible with the idea that DNAJB6 has cochaperone functions related to multiple pathways. Some experimental evidence indicates that DNAJB6 may promote proteasomal degradation of clients [30,52], and this is supported by its interaction with the proteasome subunit PSMD2 [2].

##### Antiaggregation and Cytoprotection

DNAJB6 belongs to a DNAJB subfamily characterized by potent antiaggregation activity and it is, together with its close homolog DNAJB8, probably the most efficient of human JDPs in this respect [30]. Consequently, it has been suggested to protect cells from the aggregation of protein fragments generated in catabolic processes [53].

The best-characterized clients of DNAJB6b are polyglutamine (polyQ)-containing proteins and peptides, and amyloid-β42 (Aβ42), whose amyloid aggregation DNAJB6b efficiently suppresses in vitro and in vivo [11,30,47,50,53,54,55]. This antiaggregation activity is an intrinsic property of DNAJB6b, which is independent of HSPA [30,47,53,55]. The minor J-domain-dependent activity on polyQ-huntingtin seen in cultured cells was thought to reflect the HSPA-mediated proteasomal turnover of the client [30].

Hageman and colleagues initially identified the antiaggregation activity to depend on the S/T region (then called “SSF-SST”) [30]. The critical role of this region on polyQ and Aβ42 antiaggregation has been confirmed [53,56]. Specifically, the hydroxyl side chains of the conserved Ser/Thr residues are thought to inhibit aggregate nucleation by forming competing hydrogen bonds [56]. Consistently, DNAJB6b inhibits efficiently the primary and secondary nucleation of amyloid but is less efficient against the growth of existing aggregates [47,50,53]. The anti-amyloid activity is also evident in yeast, where DNAJB6b was shown to inhibit polyQ toxicity and cure prions in a manner independent of Hsp70 but dependent on the S/T region [57].

DNAJB6b possesses antiaggregation activity also toward other clients, and this depends at least partially on mechanisms distinct from S/T-dependent anti-amyloid activity. DNAJB6b has been shown to inhibit prion-like aggregation of TDP-43 (TARDBP, transactive response DNA binding protein 43 kDa) to nuclear stress bodies upon heat shock in a partially J-domain-dependent manner [58]. Along the same lines, overexpression of the *Drosophila* DNAJB6 ortholog dMRJ suppressed cytoplasmic prion-like aggregation of mutant Hrb98DE, which is a *Drosophila* ortholog of human hnRNPAs [59].

Similar to several other cytosolic DNAJs, DNAJB6b was shown to reduce both the aggregation and steady-state levels of parkin p.C289G mutant in a cell model [31,60]. While some of this activity seemed to be HSPA-independent, as demonstrated by HSPA1 knockdown and pharmacological HSPA inhibition, the full effect was disrupted by J-domain inactivation or deletion [31,60]. Using DNAJB8, the effect was also demonstrated to be independent of the S/T region, indicating a mechanism totally distinct from polyQ antiaggregation [31].

The antiaggregation effect of DNAJB6b on α-synuclein in cells and in vitro was shown to be HSPA- and JD-dependent and largely independent of the hydroxyl groups of the S/T region [61]. Very recently, further studies confirmed the increased susceptibility of DNAJB6-deficient cells to seeded α-synuclein aggregation and suggested that DNAJB6 promotes proteasomal turnover of α-synuclein [52].

It is of note that while DNAJB6b efficiently prevents aggregate formation, it is not able to dissolve existing polyQ aggregates in cultured cells [30]. This is in line with the fact that the DNAJB6-like subfamily does not form DNAJA–DNAJB interclass dimers involved in HSPA-mediated disaggregation [33].

The antiaggregation activity of DNAJB6b toward several clients, many of which are clinically interesting, is reflected in cytoprotective effects observed in vivo. Brain-specific DNAJB6b overexpression inhibits inclusion formation, delays disease, and improves motor function in a mouse model of Huntington’s disease [53]. Likewise, in *Drosophila*, the neuronal overexpression of dMRJ or human DNAJB6b protects from polyQ-induced cytotoxicity [54,62] and, remarkably, astrocytic DNAJB6b expression also provided non-cell-autonomous protection against neuronally expressed polyQ [62].

DNAJB6b may protect cells against polyQ toxicity independently of its antiaggregation capacity, as some studies have dissociated cytoprotection from aggregate formation [11,54,63]. This could indicate that the co-aggregation of DNAJB6b modifies the aggregate structure or reflect a decreased abundance of toxic soluble preamyloid oligomers [54,63]. As suggested by Li et al. [64], a cytoprotective effect could be mediated by myeloid leukemia factors (MLF1 and MLF2), which have been shown to interact with DNAJB6 [2,64,65] and to modify the structure and toxicity of polyQ aggregates [66,67]. Notably, in *Drosophila*, the complex of MLF and DnaJ-1 plays a role in transcriptional regulation [65,68], suggesting that also in mammals, the functions of MLF1/2 with DNAJB6 could be diverse.

##### Cytoskeletal Maintenance

The interaction of DNAJB6b with keratin 18 (KRT18), and defects of the keratin cytoskeleton associated with DNAJB6b overexpression or deficiency have indicated that DNAJB6b plays a role in the maintenance of the keratin filaments [43,44]. DNAJB6 was proposed to mediate the proteasomal turnover of keratin [44], but its function could also be related to cycling of keratin subunits.

In skeletal muscle, the major intermediate filament (IF) is desmin, which attaches adjacent myofibrils at the Z-disc level and links them to the sarcolemma, mitochondria, and myonuclei [69]. Keratins 18 and 19 seem to assemble with desmin to the same IF networks, where their amount is clearly lower yet functionally significant [70,71]. The localization of DNAJB6 to Z-discs [14,49], together with the myofibrillar pathology resulting from both desmin and DNAJB6 mutations [72,73], is compatible with a role related to desmin or keratin filaments in muscle. However, an interaction with DNAJB6b and desmin was not seen in two-hybrid and cosedimentation studies [43]. Kedia and colleagues recently demonstrated that desmin contains amyloidogenic regions, and its aggregation to cytotoxic amyloid is promoted by desminopathy mutations [74]. In the light of the anti-amyloid function of DNAJB6b discussed above, an interesting possibility is that DNAJB6b serves to inhibit the seeding of desmin amyloids in the Z-disc.

Whereas DNAJB6b is associated with the IF cytoskeleton, recent research has demonstrated for DNAJB6a a role in microtubule organization during mitosis [75,76].

##### DNAJB6a in ER Stress Protection

An unexpected role in the ER was recently demonstrated for DNAJB6a [41]. In addition to the known intranuclear localization, Ding and colleagues saw perinuclear DNAJB6a-GFP localization in zebrafish heart and detected endogenous DNAJB6 at the nuclear envelope in murine heart and cultured cardiomyocytes [41]. In H9c2 cells, ER stress induced by tunicamycin promoted a punctate colocalization of DNAJB6 with the ER chaperone HSPA5 (Grp78/BiP) [41].

Zebrafish deficient for the DNAJB6a orthologue showed increased cardiac ER stress, whereas DNAJB6a overexpression inhibited ER stress in zebrafish and protected mice from doxorubicin-induced cardiomyopathy [41]. Of the DNAJB6a-specific variants identified in human cardiomyopathy patients, p.S316W was defective against ER stress and cardiomyopathy in zebrafish studies, indicating that DNAJB6a mediates clinically relevant protection against ER stress in the heart [41].

The molecular mechanism of this protective effect is not known, but it could depend on the intrinsic antiaggregation activity of DNAJB6a and/or a cochaperone function for HSPA5. It is also completely unknown what determines DNAJB6a localization (intranuclear/NE/ER) and how this relates with the different reported functions of the isoform.

##### Signal Transduction and Gene Regulation

In addition to PQC, DNAJB6 has been shown to function in signal transduction and gene regulation at multiple levels—from cell surface receptors to transcription factors and chromatin structure in the nucleus—and through a variety of molecular mechanisms [42,45,77,78,79,80,81]. Many of the affected pathways have roles in the regulation of cell proliferation and differentiation and, accordingly, DNAJB6 affects processes such as stem cell self-renewal [82] and tumorigenesis [83]. Interestingly, the functions of the two isoforms appear to be at least partially opposing. DNAJB6a suppresses malignancy [42,80,81], whereas the constitutive nuclear targeting of DNAJB6b has been shown to promote a cancerous phenotype in cell cultures [46].

As far as neuromuscular disease is concerned, the most relevant regulatory role of DNAJB6 is the activation of glycogen synthase kinase 3β (GSK3β), which has been recently implicated in the pathogenesis of DNAJB6-related myopathies (see below) [84]. DNAJB6a, in complex with HSPA8 and protein phosphatase 2A, has been shown to maintain the active dephosphorylated state of GSK3β [81], which negatively regulates both β-catenin and NFATc3 (nuclear factor of activated T cells cytoplasmic 3) pathways [85]. Recently, Findlay and colleagues demonstrated the importance of these pathways in the regulation of myogenesis. DNAJB6-deficient C2C12 myoblasts show increased GSK3β phosphorylation and concomitant increase in β-catenin and NFATc3 activity, in association with enhanced fusion and increased myotube size [84]. DNAJB6 can repress calcineurin/NFATc3-dependent gene expression also through direct interactions with NFATc3 and type II histone deacetylases (HDACs), which serve to recruit HDACs to NFAT-regulated promoters and induce chromatin remodeling [45]. While the the latter functionality was studied by Dai and colleagues using DNAJB6b constructs, the region interacting with NFATc3 and HDACs is common to both isoforms [45].

#### 2.1.3. DNAJB6 Mutations in Muscle Disease

Mutations in the *DNAJB6* gene cause dominantly inherited muscle diseases with variable clinical presentations. DNAJB6 mutations were first described in patients with dominant limb-girdle muscular dystrophy (LGMD) [14,86]. According to the revised LGMD nomenclature [87], this entity is now known as “LGMD D1 DNAJB6-related” (MIM #603511). Previously, both designations LGMD1D and LGMD1E have been used in the literature to refer to the DNAJB6-associated LGMD subtype. While most of the described DNAJB6 mutations lead to a LGMD phenotype, some mutations are associated with a distal phenotype [88,89,90].

To date, 18 pathogenic mutations have been reported in DNAJB6 (Table 2, Figure 2). Until recently, all the identified mutations clustered within a short stretch of amino acids in the G/F region, with multiple mutations affecting the same codons, highlighting the region as a mutational hot spot for muscle disease. The importance of the G/F region is further underlined by a splice site mutation that eliminates the entire domain and causes a severe, early onset disease [88].

The first unequivocally pathogenic mutations in the J domain of DNAJB6 were recently described by our group [90]. The p.A50V and p.E54A mutations are both located in the α3 helix, which according to the recently described structure is in direct contact with the α5 helix of the G/F region [28,90].

In the cohort of 48 French patients with protein aggregate myopathy, six (12.5%) had a mutation in DNAJB6 [91]. In the large-scale study of Nallamilli and colleagues, *DNAJB6* mutations accounted for 3% of molecular diagnoses in a cohort of 4656 LGMD patients from the U.S. [92]. Notably, 13 novel *DNAJB6* missense variants, located throughout the gene, were identified in the same patient cohort [92]; functional studies would be required to evaluate their pathogenicity.

#### 2.1.4. Clinical and Pathological Features

As evident from Table 2, there are clinical differences between the patients with the various DNAJB6 mutations. Most mutations cause classical adult late onset LGMDs, but some mutations (e.g., p.F91I and p.F91L) are associated with an earlier onset and much more severe pathology, whereas others (e.g., p.D98del and p.F100V) show a distal onset [88,95,97]. Moreover, inter- and intrafamilial variability may be considerable [14,73,96].

However, on the tissue level, all described DNAJB6 mutations result in similar changes characterized by protein accumulations and the aggregation of several Z-disc proteins, leading to the pathological classification as myofibrillar myopathy (MFM). The human pathology is recapitulated in the transgenic mouse model expressing DNAJB6b p.F93L [49]. Early changes are central myofibrillar lesions and Z-disc streaming that proceed to severe myofibrillar disintegration, and at later stages, autophagic rimmed vacuoles can be observed [73]. The protein accumulations in human and mouse muscles may be positive for structural proteins (desmin, myotilin, α-actinin, keratin 18) [14,49,88], RNA-binding stress-granule proteins (hnRNPA1, hnRNPA2/B1, TIA1) [49,89,90], TDP-43 [86,88,90], as well as chaperones and cochaperones (HSPA8, CRYAB, HSPB8, SQSTM1, BAG3, STUB1) [14,88,89,95,98]. The rimmed vacuoles are positive for SQSTM1 and the autophagosome marker LC3 (microtubule-associated proteins 1A/1B light chain 3), illustrating their autophagic origin [14,88,95]. The vacuoles do not stain for the lysosomal marker LAMP2, suggesting problems with autophagosome–lysosome fusion [73].

Dysphagia has been reported with several mutations [88,89,90,94,95,97,101,102,103]. Respiratory involvement is rare, but patients with the severe p.F91I and p.F91L mutations had respiratory failure requiring mechanical ventilation [95]. On the other hand, the p.F91V mutation is not apparently affecting respiration and has a much milder phenotype and progression [96]. So far, there has been no report of cardiomyopathy in DNAJB6 patients, but given the proposed role of DNAJB6a in cardiomyopathy [41], monitoring of heart function is recommended.

Despite the prominent expression of DNAJB6 in the CNS [11,41], neurological involvement is not a part of the phenotype in LGMD D1 patients. One single case of frontotemporal dementia in a p.F93L patient has been reported [104], which could just be a coincidental “double trouble” finding.

#### 2.1.5. Pathomechanistic Effects of DNAJB6 Mutations

Studies utilizing in vitro systems and model organisms have revealed functional consequences of disease mutations and offered some insight into the molecular pathomechanism of DNAJB6-related myopathies (Table 3).

##### Altered Antiaggregation Function

**Figure 3:**
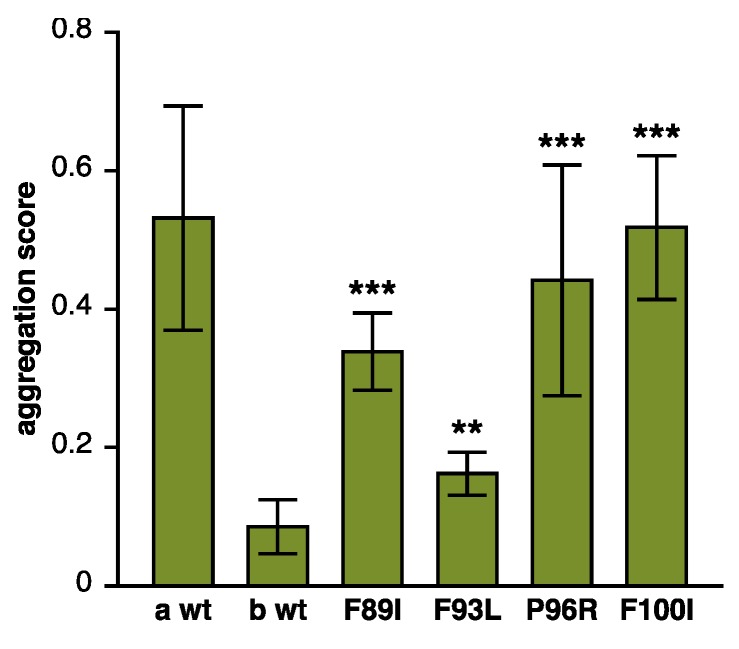
Loss of antiaggregation effect due to DNAJB6 mutations. Various DNAJB6b constructs were tested in a filter trap assay for their ability to prevent the aggregation of polyQ huntingtin. Similarly to the previously tested p.F89I and p.F93L, both p.P96R and p.F100I mutations showed impaired antiaggregation activity. Wild-type DNAJB6a (a wt) and DNAJB6b (b wt) serve as negative and positive controls, respectively. The graph shows mean ± S.D. of eight to nine replicate transfections from three separate experiments. Asterisks indicate statistically significant differences to b wt according to the Mann–Whitney *U* test (** *p* < 0.01, *** *p* < 0.001).

First of all, disease-causing mutations have been shown to impair the antiaggregation function and/or other activities of DNAJB6 toward different client proteins in a variety of experimental systems (Table 3). Most of the mutations have been studied by filter trap assay (FTA), where they consistently impair the ability of DNAJB6b to suppress the aggregation of polyQ-containing huntingtin constructs [14,89,95,102]. We report here the results for two mutations (p.P96R and p.F100I) for which FTA data have not been previously published (Figure 3). The severity of the antiaggregation defect varies greatly in this experimental system, with no clear correlation to the clinical phenotype [14,89,95,102]. The coexpression of DNAJB6b p.P96L was also shown to interfere with the antiaggregation effect of the wild-type protein, demonstrating a dominant negative effect for the mutation [102]. Moreover, the antiaggregation function of DNAJB6b toward parkin p.C289G, which depends on a molecular mechanism distinct from polyQ antiaggregation, was also somewhat impaired by the p.F93L mutation [31].

Stein and coworkers elegantly studied the effects of DNAJB6 mutations in a yeast system. In these experiments, mutations corresponding to DNAJB6 p.F89I and p.P96R, when engineered into DNAJB1, failed to complement the yeast JDP Sis1, while p.F93L was functional [105]. They also demonstrated that the myopathy-associated mutations, in the context of a Sis1/DNAJB6 hybrid protein, differentially affect the propagation and solubility of the yeast *[RNQ+]* and *[PSI+]* prions, with effects depending on prion strain and mutation in question [105]. The results demonstrated that the disease mutations specifically affect the processing of some conformers of client proteins [105].

The effects of DNAJB6 mutations on the prion-like proteins gained further support from TDP-43: DNAJB6b mutant constructs enhanced the formation of nuclear TDP-43 aggregates upon heat shock and impaired their clearance after stress, and this was also seen in fibroblasts of LGMD D1 patients [105]. Along the same lines, while the wild-type *Drosophila* DNAJB6 ortholog dMRJ was shown to inhibit the aggregation of mutant hnRNPA2 in fly muscles; the mutations corresponding to p.F89I and p.F93L prevented this effect [59]. Mutant dMRJ corresponding to p.F89I also failed to inhibit the cytoplasmic translocation of the *Drosophila* RNA-binding protein Hrb98DE to stress granules upon heat shock, and they showed reduced interaction with Hrb98DE in a pull-down assay [59].

##### DNAJB6 Turnover

Disease mutations have been shown to decrease the turnover rate of DNAJB6b in vitro cycloheximide- or de-induction-based chase assays [14,49], and this turnover difference is reflected in the increased steady-state levels of the mutant proteins in cell cultures and transgenic mouse muscles [14,49]. In line with its dominant toxic effect, mutant DNAJB6b also decreased the turnover rate of the coexpressed wild-type protein [14].

The turnover process affected by the mutations depends on the autophagy–lysosome pathway, as demonstrated by its response to lysosomal inhibition [14], but whether it represents codegradation of DNAJB6 with autophagic substrates or some other type of turnover is not known. The relationship of the altered turnover and disease pathomechanisms is also unknown, although the increased level of the mutant protein could contribute to the altered GSK3β signaling in the DNAJB6 F93L mouse model [84] (see below). Of note, the p.F93L mutation also decreased the turnover of the DNAJB6a isoform in cycloheximide assay [14], suggesting that the turnover difference is not the main factor driving pathogenicity.

##### Dominant Toxicity

Independent lines of evidence indicate that the pathogenesis depends on a dominant toxic effect mediated by the DNAJB6b isoform. In zebrafish embryos, the expression of different mutant DNAJB6b constructs has been shown to have a myotoxic effect that is evident as breakage and detachment of muscle fibers, while mutant DNAJB6a or wild-type constructs for either isoform have no such effect [14,97]. Similarly, the transgenic mouse model overexpressing human DNAJB6b p.F93L in skeletal muscle develops muscle weakness by the age of two months, whereas the corresponding DNAJB6a-expressing model does not [49]. Interestingly, the muscle defect caused by mutant DNAJB6b in zebrafish is aggravated by the equimolar coexpression of wild-type DNAJB6b, but it is rescued by a further increase in the wild-type/mutant ratio [14]. This is compatible with a model where the presence of mutant monomers in the oligomeric DNAJB6 complex, if exceeding a certain proportion, confers toxicity to the entire complex.

The recently published protein structure and the model of the DNAJ–HSPA cycle [28] suggest a possible mechanism for the toxic effects of DNAJB6 mutations. All the mutations in the G/F region are clustered in or near the α5 helix, whereas the two recently identified J-domain mutations are located close to the JD/α5 interface (Figure 2) [28,90]. The mutations may hence disturb the interaction of the α5 helix with the JD, interfering with DNAJB6 autoinhibition and leading to uncontrolled interactions with HSPA.

An interesting parallel comes from the *E. coli* DnaJ protein. The G/F region of this DNAJB6 ortholog contains three DI/VF motifs, whose counterpart in human DNAJB6 is the single motif located in the α5 helix [28]. Mutations in the DnaJ DI/VF motifs have a dominant toxic effect on bacterial growth [27]. The toxicity is dependent on DnaK (HSPA) and rescued by overexpression of the nucleotide exchange factor GrpE, and it was therefore proposed to result from kinetically trapped complexes of DnaK with DnaJ and/or client proteins [27]. The toxicity of mutant DNAJB6 could conceivably depend on similar mechanisms.

Interestingly, the cochaperone BAG3 (discussed below in more detail) was implicated in the pathomechanism of DNAJB6 mutations by the finding that the coexpression of wild-type BAG3 but not the myopathy-linked p.P209L mutant exacerbated the toxicity of mutant DNAJB6b in zebrafish [14]. While this suggests that BAG3 plays an active role in the pathomechanism, its precise place in the picture remains unknown. One possibility is that the ability of BAG3 to augment DNAJB6 toxicity is related to modulation of the HSPA ATPase cycle, as the p.P209L mutant has been shown to be defective in this respect [106]. Alternatively, the toxic effect of the stalled DNAJB6/HSPA complexes could depend on the recruitment of BAG3.

##### GSK3β Signaling

Recent results from the Weihl laboratory have indicated that the pathogenic effects of DNAJB6 mutations are partially mediated by enhanced GSK3β signaling [84]. In the mouse model expressing DNAJB6b p.F93L in skeletal muscle [49], Findlay and colleagues found dramatically reduced GSK3β Ser-9 phosphorylation, i.e., an opposite effect compared to that seen in DNAJB6-deficient myoblasts [84]. Treating the animals with lithium chloride, an inhibitor of GSK3β, improved the muscle size, strength, and myopathology, without reversing the accumulation of sarcomeric and RNA-binding proteins in mutant muscle [84].

The molecular mechanism through which the DNAJB6b p.F93L mutation exerts its effect on GSK3β signaling is unclear, but increased level of the mutant protein was proposed as one possible explanation [84]. Interestingly, while the isoform originally linked to GSK3β was DNAJB6a [81], these new findings suggest that both DNAJB6 isoforms may participate in GSK3β signaling and raise intriguing questions on the roles of the isoforms in the pathomechanism of LGMD D1. Although direct comparison of transgenic lines with different DNAJB6 expression levels has its limitations, according to Bengoechea et al., the overexpression of DNAJB6a p.F93L does not cause muscle weakness in mice [49]. This would mean that either the disease mutations do not affect GSK3β signaling in the context of the DNAJB6a isoform or that a DNAJB6b-specific effect combined to altered GSK3β signaling is required to mediate the pathogenesis. To address these possibilities, it would be useful to review the status of GSK3β signaling in the DNAJB6a p.F93L model.

**Figure 4:**
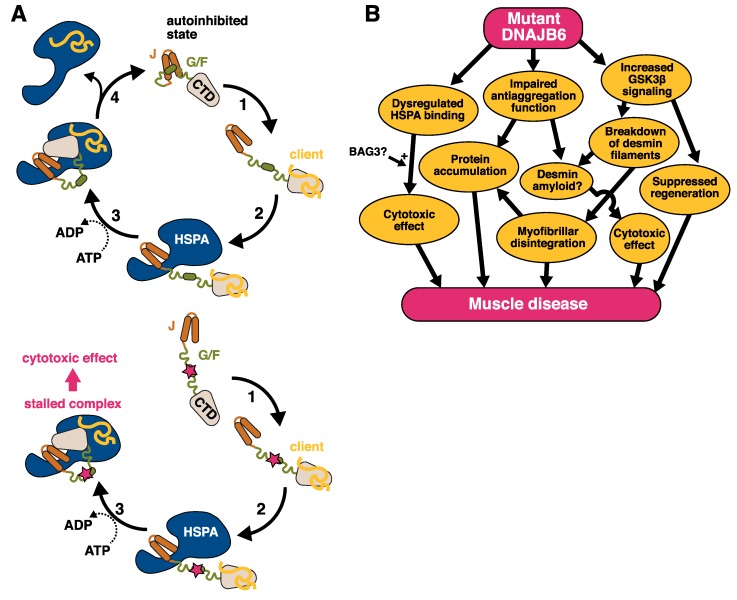
The pathomechanism of DNAJB6 mutations. (**A**) Top: The suggested function of DNAJB6 in the HSPA cycle: (1) Client binding by DNAJB6. (2) HSPA binding to JD. (3) ATP hydrolysis and client transfer to HSPA. (4) Displacement of HSPA by the G/F domain. Adapted from [28]. Bottom: A model for the effect of myopathy mutations. The mutations interfere with the interaction between the J and G/F domains, leading to uncontrolled interaction with HSPA. (**B**) Possible parallel downstream pathways leading from DNAJB6 mutations to muscle disease.

Overall, functional studies have demonstrated that disease-causing mutations affect DNAJB6 function in multiple ways. Yet, more research is needed to elucidate the causal relationships between these effects and their importance in the pathogenesis of DNAJB6-related myopathies. It seems likely that the accumulation of various proteins in diseased muscle reflects the defective processing of client proteins, but it remains unknown whether the aggregation pathology is driven by one or a few selected clients or a general impairment of chaperone function. Another open question is if the toxicity of mutant DNAJB6 simply results from a dominant negative effect on the wild-type allele, or if the mutant DNAJB6 complexes have additional toxic properties. In any case, the available data are compatible with a scenario where DNAJB6 mutations cause disease through two or more parallel mechanisms (Figure 4): The loss of protective chaperone effect and/or active toxicity of the mutant DNAJB6 damage the myofibers, and at the same time abnormal GSK3β signaling interferes with muscle regeneration. Interestingly, GSK3β was recently shown to promote the breakdown of desmin filaments in muscle atrophy [107], providing another mechanism potentially contributing to the disease.

An intriguing question still lacking explanation is the specific pathogenic effect of mutant DNAJB6 in skeletal muscle. Although DNAJB6 is highly expressed in the CNS [11], brain symptoms are not a typical feature in DNAJB6-related diseases. Differences in protein expression are naturally a possible explanation for the tissue selectivity. Muscle may express high levels of a key client protein whose aggregation drives the pathogenesis or factors modulating the toxicity. One factor possibly contributing to the tissue selectivity could be BAG3, which is highly expressed in muscle [108] and known to augment the toxicity of mutant DNAJB6b in zebrafish [14].

### 2.2. DNAJB2

**Figure 5:**
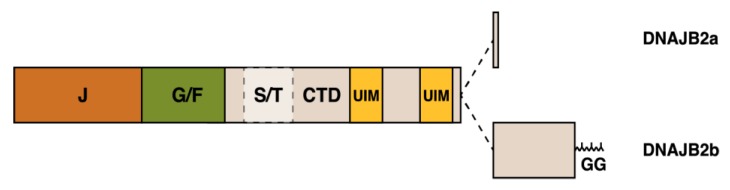
Schematic structure of the DNAJB2 protein and the alternatively spliced C-terminal parts of the two isoforms. The J, G/F, and C-terminal (CTD) domains, the Ser/Thr-rich region, two ubiquitin-interacting motifs (UIM) and the C-terminal geranylgeranyl (GG) anchor of DNAJB2b are indicated.

DNAJB2, first described as HSJ1 [109], belongs to the class II (DNAJB subfamily) of J proteins and, accordingly, contains an N-terminal J-domain followed by a G/F region [110] (Figure 5). As a unique feature among human J proteins, the C-terminal region of DNAJB2 harbors two ubiquitin interaction motifs (UIMs) that mediate binding to polyubiquitylated proteins and to the proteasome [21,111]. Alternative splicing produces two isoforms that differ in their C-termini and show different subcellular localization: DNAJB2a (HSJ1a; 277 aa, 31 kDa) localizes to the cytosol and nucleus [110], whereas DNAJB2b (HSJ1b; 324 aa, 36 kDa) is associated to the cytoplasmic face of ER by a C-terminal geranylgeranyl anchor [110].

#### 2.2.1. DNAJB2 Expression

DNAJB2 is predominantly expressed in neurons, with the highest expression levels seen in the neocortex [109,110]. A low level of the protein has been detected in other cells and tissues, as well as in fibroblast cultures [110,112,113]. The clearly predominant isoform in neuronal tissues is DNAJB2b [110,112,113,114].

Low DNAJB2 expression in cardiac and skeletal muscles was reported by Claeys and colleagues, who saw DNAJB2 localized to the neuromuscular junction in mature muscle fibers and to the sarcoplasm and sarcolemma in regenerating fibers [115]. However, studies on *Dnajb2*-deficient mice indicate that the neuromuscular junction localization reported by Claeys et al. may be due to the cross-reactivity of the commercial antibody used in that study (Michael Cheetham, personal communication). Nevertheless, upregulation of *DNAJB2* mRNA after eccentric exercise [116] supports the notion of physiologically relevant DNAJB2 expression in muscle.

#### 2.2.2. Functions of DNAJB2

As expected for a JDP, DNAJB2 acts as a cochaperone for HSPA: both isoforms have been shown to stimulate the ATPase activity of HSPA8 and to modulate its client binding [10]. Instead of promoting the refolding of HSPA clients, DNAJB2 is considered to primarily direct them to degradation by the ubiquitin–proteasome system (UPS) [111,117]. To this end, DNAJB2 promotes the ubiquitylation of client proteins by STUB1 (CHIP) and, by binding to the polyubiquitin chains, it protects them from deubiquitylation [111]. Then, HSPA-bound ubiquitylated clients are targeted to the UPS [111,117], which may be facilitated by the ubiquitylation of DNAJB2 itself and the DNAJB2-stimulated ubiquitylation of HSPA [111]. The binding of ubiquitylated clients is negatively regulated by the phosphorylation of UIM2 by protein kinase CK2 [118]. In addition to HSPA, DNAJB2 may act together with HSPC (Hsp90). This is suggested by the interaction of the chaperones in vitro and their ability to transfer client proteins between each other [119].

DNAJB2 has been demonstrated to suppress the aggregate formation of various client proteins in different experimental systems. These effects depend on—as deduced from the effects of deletion and mutant constructs—a combination of UPS-mediated degradation and other mechanisms [30,31,60,111,112,114,117,120,121,122,123]. Indeed, as demonstrated by its ability to suppress luciferase aggregation in vitro, DNAJB2 has intrinsic chaperone activity that resides outside the J–G/F-region and is independent of functional UIMs [111]. As DNAJB2 contains a short S/T-rich region C-terminally from the G/F region—including the Ser/Thr residues most critical for DNAJB6 client binding [53,56]—this region could be involved in the intrinsic chaperone function.

DNAJB2a has been in several studies shown to suppress aggregates of polyQ-containing huntingtin and androgen receptor both in vitro [30,111,114,117] and in vivo [117,120] in a manner dependent on J domain and UIMs [111,120]. DNAJB2a also reduced the aggregation and steady-state levels of parkin mutant p.C289G, similarly to other cytosolic DNAJs [31,60]; this effect was partially dependent on HSPA [31,60], but it did not require UIMs [60]. DNAJB2 counteracted the aggregation of SOD1 p.A4V and p.G93A mutants in vitro [112,121] and improved the disease phenotype in the SOD1 p.G93A mouse model of familial ALS [121]. In cell culture, SOD1 p.G93A ubiquitylation was UIM-dependent, whereas the J-domain mutant p.H31Q promoted SOD1 ubiquitylation but failed to mediate its turnover [121]. Recently, DNAJB2a was also shown to decrease the aggregation of overexpressed TDP-43 in a cell model [122]. In contrast to most other reported DNAJB2 functions, this effect was independent of UIMs and UPS-mediated degradation but reflected refolding of TDP-43 in a J-domain-dependent fashion [122].

While the effects of DNAJB2b against cytoplasmic and nuclear protein aggregation have been variable [30,31,111,114], a number of studies indicate that the physiological functions of this membrane-bound isoform are related to the quality control, handling, and degradation of secreted and transmembrane proteins. DNAJB2b has been demonstrated to modulate rhodopsin processing in neuroblastoma cells [110], to facilitate the proteasomal degradation of cystic fibrosis transmembrane conductance regulator (CFTR) through the ER-associated degradation (ERAD) pathway [111], and to reduce the total and cell-surface levels of melanocortin 4 receptor (MC4R) in a cotransfection setup [124]. DNAJB2 was also found to promote the lysosomal targeting of misfolded CFTR p.F508del from the plasma membrane [125], although the isoform involved in this function was not determined.

Both DNAJB2 isoforms can inhibit HSPA-mediated uncoating of clathrin-coated vesicles in vitro [126]. While this was suggested to reflect interference with another J protein such as auxilin [126], the work of Borrell-Pagès and coworkers later demonstrated that DNAJB2b specifically promotes the sorting of clathrin-coated vesicles from the Golgi apparatus [114]. This is important for the secretion of brain-derived neurotrophic factor (BDNF)—and possibly other proteins—from the Golgi and has implications on Huntington’s disease, where the process is impaired due to decreased DNAJB2b levels [114].

#### 2.2.3. DNAJB2 Mutations in Neuromuscular Disease

Recessive *DNAJB2* mutations (Table 4) have been identified as a so far uncommon cause of peripheral neuropathies, which may present as distal hereditary motor neuropathy (dHMN) or sensory and motor neuropathy (Charcot–Marie–Tooth disease type 2, CMT2) [112,113,127,128,129].

*DNAJB2* was first associated to disease by Blumen and colleagues [112], who identified a homozygous c.352+1G>A splice donor site mutation in a family with dHMN. The same mutation, and another recessive splice site change c.229+1G>A have since been reported in several families [113,128]. These mutations have been shown to lead to intron retention, premature termination, and severely reduced or lost DNAJB2 protein expression in patient fibroblast cultures [112,113]. The mutation c.619-1G>A, abolishing a splice acceptor site, and the single-nucleotide deletion c.310delC, leading to cause frameshift and premature protein termination, have been found in homozygous state in individual families [37].

A large (approximately 3.8-kb) homozygous deletion spanning the first four exons of *DNAJB2* end extending approximately 1.3 kb upstream of the gene was identified by Sanchez et al. [129] in a family with dHMN in two siblings and additional juvenile parkinsonism in one sibling. Involving the first exon of *DNAJB2*, this deletion likely results in a null allele similarly to the reported splice site mutations.

The only published missense mutation reported so far in *DNAJB2* is c.14A>G (p.Y5C), which was identified in homozygous state in a single family with a CMT2 clinical phenotype [113]. The mutation affects the J domain, substituting a tyrosine residue conserved in J proteins throughout evolution [113]. Functional consequences of this mutation have not been reported, but segregation and prediction algorithms support its pathogenicity [113]. The variant is also not found in gnomAD (The Genome Aggregation Database).

#### 2.2.4. Clinical Features of DNAJB2-Related Neuropathies

The main clinical phenotype resulting from the biallelic loss of *DNAJB2* expression is peripheral axonal neuropathy. The onset of symptoms is typically in the 2nd decade of life, and has been reported to range from the late 1st to early 4th decades [112,113,127,128,129]. The initial diagnosis may be pure motor neuropathy (dHMN) [112,113], which manifests as pareses, muscle weakness, and atrophy appearing first in distal lower limbs and progressing slowly to proximal lower limbs and arms [112,113,127,128,129]. Bulbar and respiratory symptoms may develop at the advanced stage [128]. Sensory symptoms such as decreased sensation appear with age in many if not all patients [127,128,129]. In terms of clinical findings, the p.Y5C missense change seems comparable with the *DNAJB2* null mutations [113].

Symptoms involving the central nervous system have been described in some patients with *DNAJB2* mutations. Early-onset parkinsonism has been reported in a few patients from different families [128,129,130], whereas frontotemporal brain atrophy with behavioral changes [128] and cerebellar ataxia [130] have been seen in individual patients. Due to the small number of cases, it remains unclear whether the CNS symptoms in these patients are indeed due to the *DNAJB2* mutations or additional factors [128].

#### 2.2.5. Pathomechanisms of DNAJB2 Mutations

The splice site mutations c.229+1G>A and c.352+1G>A have been demonstrated to disrupt *DNAJB2* expression in fibroblasts [112,113], and they presumably have the same effect in neurons. In addition, c.619-1G>A and c.310delC are expected to result in a loss of DNAJB2 expression. The pathogenesis of recessive DNAJB2-related neuropathies is hence most likely to depend on a loss-of-function mechanism [113,129,131]. The client protein(s) and processes relevant for the disease remain to be established: the pathomechanism could be envisioned to depend on cytotoxicity due to impaired protein quality control and turnover or a specific defect in protein trafficking or secretion caused by loss of DNAJB2b.

As the 3.8-kb deletion described by Sanchez et al. spans the *DNAJB2* exons encoding the J domain, the authors utilized a GFP-DNAJB2b construct lacking the J domain to study the functional effects of the deletion [129]. When expressed in HEK-293 cells, this construct showed aggregation and induced cell death, which was accompanied with increased LC3 expression [129]. The mutant construct was also reported to increase the expression of the DNAJB2a isoform on both RNA and protein levels, as well as alter Tau expression and BDNF release [129], although the data presented in the paper do not exclude alternative interpretations. Nevertheless, the apparent toxicity of N-terminally truncated DNAJB2b may not in this case be pathomechanistically relevant: As also suggested by its recessive inheritance, the deletion most likely totally prevents *DNAJB2* expression and is hence comparable to the splice mutations. Moreover, the deletion also affects a predicted isoform of the *TUBA4A* gene, mutations in which are associated with ALS [132], and this could also contribute to the clinical phenotype.

In the absence of functional data, the pathomechanism of the DNAJB2 p.Y5C mutation can only be speculated. Given its recessive inheritance and the phenotype comparable to the splice mutations, the mutation is likely to produce a loss-of-function allele. In line with this, structural data supports the idea that the variant could destabilize the J domain (Per Harald Jonson, unpublished observation).

## 3. Small Heat Shock Proteins

The small heat shock proteins (sHSP) are an ancient group of molecular chaperones that are present in all kingdoms of life [4]. Of the 10 sHSPs (or HSPBs) encoded by the human genome [23,133,134], four are currently known to be associated with neuromuscular disease [9]; these are HSPB1 (Hsp27), HSPB3, αB-crystallin (CRYAB, HSPB5), and HSPB8 (Hsp22) (Table 1).

### 3.1. Structure and Function of sHSPs

The defining structural feature of sHSPs is the α-crystallin domain (ACD) that plays a key role in client binding and mediates the dimerization of sHSPs into homo- or heterodimers. The ACD is flanked by N- and C-terminal extensions that mediate the chaperone activity and are responsible for the functional specificity of the family members [135]. The N-terminal domains (NTD) are long, hydrophobic, in part intrinsically disordered or quasi-ordered (i.e., alternating between several defined states), and highly variable between the different sHSPs, whereas the C-terminal domains (CTD) tend to be polar and rather short [136,137,138].

Canonical sHSPs assemble further into large homo- and hetero-oligomeric complexes; this is driven by binding of the ACD to the IxI/V motif present in the CTD, as well as NTD–ACD and NTD–NTD interactions [138,139,140,141]. The architecture of the oligomeric complexes differs among the family members. While some sHSPs form discrete oligomers, others—such as those formed by HSPB1 and CRYAB—are polydisperse and highly dynamic [17,142,143,144].

The canonical function of small heat-shock proteins is to act as the cell’s first-line response against non-native proteins. sHSPs have often been described as “holdases” that bind partially unfolded proteins and protect them from aggregation, keeping them available for refolding or degradation by other chaperone families [4,137]. However, rather than keeping clients in a soluble state, sHSPs complexed with their clients are often found to coaggregate with them. This prevents cytotoxicity of the aggregates and facilitates later recovery and refolding or degradation of the aggregated proteins [145,146,147,148]. Moreover, as best characterized with yeast Hsp42, sHSPs may even function as “sequestrases” that actively drive the controlled aggregation of non-native proteins, thereby preventing the overburdening of the refolding machinery during cellular stress [148,149,150,151,152,153].

While the sHSPs system is energy-independent and has a high client-binding capacity, allowing an efficient first-line protection against unfolded proteins, sHSPs lack the ability to actively refold clients [154,155,156]. For refolding or degradation, the client proteins stably complexed with sHSPs are extracted by HSPA chaperones, potentially with the assistance of JDPs [154,155,157]. The cooperation of sHSPs and HSPAs is facilitated by the cochaperone BAG3 that interacts with HSPAs [158] and several sHSPs, providing a physical link between the chaperone families [2,15,16,18,159].

The interactions of sHSPs with their client proteins are complex: depending on the type of client (e.g., amorphous versus amyloid aggregates) and the stage of protein aggregation, the interactions can be stable “holdase”-type or transient, and involve different regions of the protein [144,160,161,162,163,164,165,166,167]. Some client interactions are mediated by the ACD, whose local unfolding and dimerization status can, even in the context of the oligomeric complex, regulate the availability of client-binding surfaces [168,169,170,171]. Other client interactions depend on the extensions, especially the NTD, whose disordered nature is thought to facilitate binding to a diverse spectrum of client structures [136,144,160,162].

The relationship of sHSP oligomerization and chaperone activity has been the subject of extensive research, with partly contradictory results obtained with different sHSPs and clients [161,165,172,173,174,175,176,177,178]. In some setups, mutations or modifications favoring deoligomerization have been associated with increased chaperone activity [161,165,177], while others have shown an opposite effect [161,172,173]. Moreover, oligomer dissociation and reassembly have been found to be variably required for chaperone function [174,175,176,178]. These divergent results likely reflect the functional complexity of sHSPs, with different binding modes being important for different clients, as well as genuine differences between family members.

Overall, the current picture is that the dynamic oligomeric architecture allows, by regulating the accessibility of the client-binding surfaces, sHSPs to flexibly utilize different binding modes while protecting them from inappropriate interactions and aggregation [17,166,167,171,179,180]. The equilibrium between monomers, dimers, and oligomers, and hence the chaperone activity, can be regulated by phosphorylation [138,161,167,169,172,176,181,182,183,184] and stress-related environmental factors such as temperature [185,186], pH [179,187], metal ions [188], and the redox state [171]. The system can be fine-tuned by hetero-oligomerization, which is thought to offer an optimal combination of stability and chaperone activity [17,180]. In addition, BAG3 binding has been recently shown to dissociate HSPB1 oligomers, allowing it to modulate sHSP function [16]. Notably, some sHSPs with non-canonical modes of action, e.g., HSPB8 discussed in more detail below, do not form large oligomers [189,190].

In the following sections, will review the neuromuscular diseases caused by sHSP mutations, focusing on the current understanding of the pathomechanisms. A concise overview of the functions is provided for each of the proteins. The disease-associated sHSPs—especially CRYAB and HSPB1—have been the subject of extensive research, and covering their vast range of normal functions is not possible here. For additional details, we refer the reader to recent reviews [4,137,191,192,193,194].

### 3.2. αB-Crystallin (HSPB5)

αB-crystallin (CRYAB, HSPB5) is a major structural protein of the lens, but shows stress-inducible expression also in other tissues where it acts as a multifunctional chaperone [191,192]. Its levels are particularly high—up to 3% of soluble protein—in the heart and in skeletal muscle, where it shows highest expression in slow fibers [195,196,197,198]. In addition to diffuse sarcoplasmic localization, CRYAB associates with the Z-discs and I-bands and cardiac intercalated discs. The myofibrillar localization is promoted in stress situations such as ischemia, stretch, and eccentric contractions, reflecting an increased association of CRYAB with its client proteins desmin and titin [199,200,201,202,203,204].

The principal function of CRYAB is the stabilization of cytoskeletal and sarcomeric proteins [192]. It shows temperature- and pH-dependent association with desmin and other intermediate filaments, promotes filament assembly, and inhibits their aggregation [192,205,206,207,208,209,210]. In addition, CRYAB is known to chaperone actin [205,211,212,213] and tubulin [214,215,216,217]. In the sarcomeric I-band, CRYAB binds the spring elements of titin: cardiac-specific N2B unique sequence (N2B-us), the N2A element, and immunoglobulin (Ig) domains. This modulates titin elasticity, prevents the unfolding of the Ig domains, and protects them from aggregation [201,202,204,218].

Many studies have implicated CRYAB in the regulation of apoptosis [213,219,220,221]. With potential relevance for muscle disease, the upregulation of CRYAB during early myogenic differentiation protects myoblasts from apoptosis by inhibiting caspase 3 activation, and this effect was shown to be blunted by the p.R120G mutation (see below) [219].

#### 3.2.1. Neuromuscular Diseases Due to CRYAB Mutations

The first pathogenic CRYAB mutation, causing the p.R120G in the ACD, was identified in 1998 by Vicart and colleagues in a French family with dominantly inherited myofibrillar myopathy, hypertrophic cardiomyopathy, and cataracts [222]. Based on the myopathology characterized by desmin accumulation, the disease was described as a desmin-related myopathy [222] and is now classified in the Online Mendelian Inheritance in Man (OMIM) database as myofibrillar myopathy 2 (MFM2, MIM #608810).

Additional dominant missense and truncating variants (Table 5) have been identified in patients with comparable combination of phenotypes, i.e., myopathy and variable cardiac involvement, often together with cataracts [223,224,225,226,227], and in some cases with isolated cardiomyopathy [228,229]. The myopathy phenotypes show variability in age of onset and muscle involvement: the weakness may be widespread—including trunk, neck, velopharyngeal, and respiratory muscles in addition to proximal and distal limb muscles—or show a more limited distal involvement [222,223,224,225,226,227]. Neuropathy has been reported in isolated cases [223].

Pathologically, αB-crystallinopathy shows typical features of myofibrillar myopathies, with most resemblance to primary desminopathy [230,231]. Histological hallmarks are protein accumulations, which are notably positive for αB-crystallin and desmin, and vacuoles. Staining for oxidative enzymes reveals “rubbed-out fibers”, with large areas devoid of mitochondrial activity. In electron microscopy, granulofilamentous desmin accumulations, myofibrillar disorganization, with Z-disc streaming and longitudinal Z-disc extensions [72,230,231]. Changes typical to αB-crystallinopathy are “sandwich formations” where granulofilamentous material is seen sandwiched between mitochondria and frequent early apoptotic myonuclei [72,230,231].

A distinct CRYAB-related disease is fatal infantile hypertonic myofibrillar myopathy (MIM #613869), caused by recessively acting *CRYAB* mutations (Table 5). The truncating frameshift mutation p.S21Afs*24 was first found to underlie an infantile muscular dystrophy described in Canadian aboriginals [232], and two other mutations, p.S115Pfs*14 and c.3G > A (p.Met1?) have been identified in patients from other populations [233,234]. The severe congenital disease, most severely affecting truncal muscles, leads to death in infancy due to respiratory insufficiency [232,233,234]. Histopathological changes indicate a severe MFM, with inclusions of Z-disc origin and rimmed vacuoles [232,233,234].

In addition to mutations causing cataracts in combination with neuromuscular disease, several recessive or dominant *CRYAB* mutations have been associated with isolated cataracts.

#### 3.2.2. Pathomechanisms of CRYAB Mutations

The pathomechanisms αB-crystallinopathy have been thoroughly investigated in functional in vitro studies and animal models, which have demonstrated that *CRYAB* mutations lead disease through pleiotropic effects.

##### Animal Models

Much of the present knowledge is based on mouse models with cardiac-specific expression of p.R120G mutant CRYAB [235,236,237]. The “CryAB^R120G^ TG” model developed by the Robbins laboratory was based on the murine *Cryab* gene [235], whereas the “hR120GCryAB” model of the Benjamin laboratory utilized the human gene [237]. The models show some differences potentially due to different expression levels or background mouse strains, and they have been hence suggested to reflect different stages of human disease [238,239]. Nevertheless, the models have highlighted the multitude of the possible downstream effects of CRYAB mutations, many of which are likely to contribute to the pathogenesis also in skeletal muscle.

The common pathological features of the cardiac p.R120G models resemble human MFM, with desmin mislocalization, the presence of desmin and CRYAB aggregates, disrupted Z-disc structure, mitochondrial abnormalities, and fibrosis [235,237,240,241,242]. Both models develop cardiomyopathy and eventually die of congestive heart failure at 25–28 or 40–60 weeks of age, depending on the model [235,237]. A notable difference is apoptosis, which is more prominent in the CryAB^R120G^ TG model than in hR120GCryAB [238,241].

More recently, a knock-in (KI) model, expressing CRYAB p.R120G at a physiological level, was developed [243]. These mice recapitulate the human cataract and skeletal myopathy phenotypes with dose-dependent severity, but unlike the overexpression models, they do not show cardiac lethality [243].

Another relevant mouse model is the *Cryab/Hspb2* dKO, with the disruption of both *Cryab* and the neighboring *Hspb2* genes [244]. These mice develop a myopathy phenotype most prominently affecting axial and head muscles and the tongue, consistently with the highest expression of CRYAB in slow muscles, and they die prematurely presumably due to feeding difficulties [244,245,246]. The affected muscles show myopathological changes indicative of degeneration, including central nuclei, fibrosis, fatty infiltration, increased desmin staining, vacuolization, and the accumulation of amorphous material evident in EM [244], but the myofibrillar ultrastructure appears normal [244,245]. The regeneration capacity of skeletal muscles is impaired, which was suggested a dependence on altered miRNA signaling [247]. The heart appears normal in the baseline situation, although functional effects appear in stress situations [245,246,248,249].

The importance of CRYAB for muscle integrity, and the dominant pathogenic effect of the p.R120G mutation were recently also demonstrated in *Drosophila*, where the pathological changes associated with CRYAB deficiency are remarkably similar to mammalian muscle [212].

##### Structural Effects

On the structural level, p.R120G has been shown to alter the secondary and tertiary structure of CRYAB and decrease its thermal stability, causing the protein to unfold and precipitate over time [208,250,251,252,253]. Destabilizing effects have also been reported for several other CRYAB mutations (Table 5). The arginine residue affected by p.R120G is located at the dimer interface, and its substitution disrupts a salt bridge with Asp109, alters the ionic interaction network on the dimer interface, and interferes with the pH-dependent dimer dissociation that mediates chaperone activation [179,254,255]. The importance of these structural alterations is highlighted by the three disease mutations affecting the interacting Asp109 residue (p.D109H, p.D109A, and p.D109G) [225,226,227]. These are likely to have similar structural consequences as the p.R120G mutation, although different clinical phenotypes suggest some mutation-specific effects or modulation by additional factors [225,226,227].

Oligomers formed by CRYAB p.R120G are enlarged and more polydisperse compared to the wild-type protein [167,208,210,250,256], yet they show faster subunit exchange [256]. The same is seen in hetero-oligomers with HSPB1; these are larger but dissociate more easily in oxidative stress [257]. The effects of other studied mutations on CRYAB oligomerization are diverse, most are reported to cause smaller oligomers (Table 5). Here, an exception is the p.Q151* truncation, which was shown to be totally unable to oligomerize on its own and from smaller oligomers when coexpressed with wild-type CRYAB [258]. In addition, differentially altered interactions of mutant CRYAB with other sHSPs (Table 5) have been proposed to explain some phenotypic differences of the mutations [259].

##### Chaperone Activity and Client Interactions

In in vitro chaperone assays utilizing various model clients, most of the analyzed mutations have shown complex client-dependent alterations in chaperone function, with activity decreased for some clients and increased for others (Table 5) [167,208,250,251,252,253,260,261]. Pathomechanistically more clear-cut are the drastic effects of mutant CRYAB on desmin and other intermediate filaments, the physiological clients of CRYAB. In vitro, CRYAB p.R120G shows increased binding to desmin and GFAP filaments and promotes rather than inhibits filament–filament interactions [208,209,210]. In cultured cells, while wild-type CRYAB promotes the assembly of desmin filaments, p.R120G fails to do so and causes desmin aggregation, which is most severe in conditions involving IF remodeling [209,210,222]. Other IF types (vimentin and keratin) have been variably affected by CRYAB p.R120G in different experimental setups [210,262,263,264], possibly reflecting the sensitivity of IF aggregation on filament status [209]. In addition to p.R120G, a few other CRYAB mutations have been shown to increase binding to desmin filaments [258]. However, a direct pro-aggregation activity on desmin is not required for pathogenicity, as the p.Q151* mutant was found to inhibit filament–filament interactions more efficiently than wild-type CRYAB [258].

Interestingly, the findings of Elliott et al. suggested for desmin an active role in mediating the pathogenic effects of mutant CRYAB, potentially contributing to the muscle specificity [210]. CRYAB p.R120G was found to decrease the viability of transfected MCF7 cells only when cotransfected with wild-type desmin [210]. This effect, which is mimicked by expression of myopathy-causing mutant desmin alone, was thought to depend on the reported function of caspase-6-cleaved desmin as a pro-apoptotic molecule [210,265].

Besides desmin, titin is another muscle-specific CRYAB client affected by the mutations. The p.R120G and p.R157H mutations have been demonstrated to decrease the binding of CRYAB to titin N2B-us and the following Ig domains [228]. Consistently, the mutations diminish (p.R175H) or totally abolish (p.R120G) the effects of CRYAB on the extensibility of N2B-us and unfolding of Ig domains [218]. Hence, structural changes of titin spring elements may be yet another pathogenic factor in αB-crystallinopathy. Notably, p.R157H showed a more pronounced effect on the interaction with the heart-specific N2B-us, which was suggested to explain the cardiac-specific phenotype associated with this mutation [228]. Indeed, as p.R157H does not appear to cause CRYAB aggregation, its subtly altered activity toward titin could be envisioned to give rise to a unique pathomechanism leading to a mild cardiac phenotype [228].

Finally, CRYAB has chaperone functions in the nucleus as well, and their impairment may also contribute to the pathogenesis. Regulated by the phosphorylation at Ser59 and Ser45, CRYAB is imported by the survival of motor neuron (SMN) complex to nuclei, where it localizes into nuclear speckles and mitotic interchromatin granules [266,267]. Upon heat stress, CRYAB is released from the speckles to the nucleoplasm, where it can exert chaperone activity on nuclear proteins [268,269]. Through a yet incompletely understood mechanism, the p.R120G mutation interferes with the nuclear import of mutant CRYAB itself as well that of the wild-type protein, thus abolishing the protective function in the nucleus [267,268]. Cytoplasmic inclusions formed by the hyperphosphorylated mutant protein recruit SMN [267], which could also interfere with the nuclear import of small nuclear ribonucleoproteins (snRNPs) or other SMN-dependent proteins. However, it is of note that most of the above results on nuclear import and functions of CRYAB have been obtained from HeLa cells and are not entirely consistent with the findings of Adhikari et al. from C2C12 cells [270]. In C2C12 myoblasts, heat shock induced the nuclear import of CRYAB and increased its colocalization with lamin A/C in nuclear speckles, while in differentiated myotubes, stress-induced nuclear localization was not seen [270]. Hence, further studies are required for evaluating the nuclear chaperone functions of CRYAB in skeletal muscle and their role in αB-crystallinopathy.

##### Aggregation and Amyloid Formation

Propensity to aggregation in vitro and in vivo is a nearly universal feature of the analyzed CRYAB mutations (Table 5), which has been demonstrated for p.R120G [222,262,263,271,272] as well as several other mutations [227,258,259,272,273,274]. CRYAB p.R120G expressed in cells or transgenic tissues forms aggregates that coalesce into perinuclear aggresomes [262,271]. Notably, for p.R120G, aggregation has been shown to depend on the hyperphosphorylation of the NTD, which occurs both in cells and in transgenic heart [267]. A nonphosphorylatable mutant version of CRYAB p.R120G does not from inclusions and also shows normal oligomer size [267], suggesting that increased oligomer size and aggregation are mechanistically linked. While p.Q151* and p.P155Rfs*9 have also been shown to be hyperphosphorylated [259], it is not known whether this is a prerequisite for the aggregation for all the different CRYAB mutants.

The CRYAB aggregates are of amyloid nature, as first suggested by the presence of amyloid-specific staining in the aggresomes of CryAB^R120G^ TG hearts [271]. Even wild-type CRYAB is amyloidogenic in destabilizing conditions, and this property is enhanced by the structural changes caused by p.R120G and presumably other disease mutations [275,276]. In fact, p.R120G was shown not to affect the final structure of the CRYAB amyloid fibrils; in contrast, it actually delays fibril growth, suggesting its prolonged existence in the soluble preamyloid oligomer stage, which is considered to be the most cytotoxic protein species [276]. Indeed, the preamyloid oligomer rather than aggresomes has been shown to be mainly responsible for cardiac lethality in CryAB^R120G^ TG mice [236,277]. Furthermore, recombinant CRYAB p.R120G is acutely cytotoxic to cultured cells when added to media [278].

The downstream harmful effects of amyloid species may be mediated by multiple mechanisms such as membrane damage, mitochondrial dysfunction, and impairment of the UPS [279]. Indeed, CRYAB mutations have been linked to mitochondrial problems, which are briefly discussed below [239,241]. Likewise, proteasomal malfunction in cells expressing CRYAB p.R120G and in CryAB^R120G^ TG hearts is attributable to amyloid accumulation [278,280], and this may have widespread effects from cellular signaling to sarcomeric maintenance [280]. On the other hand, while aggregates are considered less toxic than preamyloid oligomers, they may hamper cellular function by the sequestration of essential proteins such as HSPB1 [257,273] and, obviously, desmin [242]. Aggregates have also been suggested to physically impair the contractility and the alter cytoskeletal properties in CryAB^R120G^ TG cardiomyocytes [239,241], although myofibrillar misalignment and the aggregation of titin spring elements may also contribute to these changes [218,239].

The clearance of insoluble aggregates depends heavily on macroautophagy. However, autophagic flux is impaired both in CRYAB p.R120G transduced cardiomyocytes and transgenic heart, which is presumably due to increased mechanistic target of rapamycin (mTOR) signaling [242,281], and its restoration has been associated with reversal of the pathogenic effects [242,281,282,283,284]. In several studies, boosting autophagy in cultured cells or in vivo has promoted the clearance of CRYAB p.R120G aggregates and/or preamyloid oligomer, resulting in improved cell viability, amelioration of cardiac pathology, and prolonged mouse survival, whereas suppressing autophagy has exacerbated the disease-related changes [238,242,281,282,283,284]. Strategies proven to be beneficial include the overexpression of Bcl-2 [282], Atg7 [281,283], or TFEB [242,284]; voluntary exercise [277,283]; and intermittent fasting [242]. Very interestingly, in a recent study, Ma and colleagues demonstrated that virally mediated overexpression of TFEB—or stimulation of TFEB activity by intermittent fasting—improved the cardiac pathology of the hR120GCryAB mice by two mechanisms: While the stimulation of autophagic flux removed CRYAB aggregates, the upregulation of HSPB8 normalized sarcomeric ultrastructure and desmin localization independently of autophagy, apparently by chaperoning the desmin molecules released from aggregates to their correct localization [242].

In addition to autophagic stimulation, other strategies successfully used to decrease mutant CRYAB aggregates and improve cardiac pathology in p.R120G transgenic mice include upregulation of the UPS by overexpression of the proteasome 28 subunit α [285] and doxycycline treatment [286].

##### Mitochondria and Redox Status

The “rubbed-out” fibers in αB-crystallinopathic muscles suggest mitochondrial depletion at an early stage [287]. In line with this, CryAB^R120G^ TG murine hearts show altered mitochondrial ultrastructure and localization, and early impaired respiratory function [241]. The mitochondrial abnormalities may be due to several upstream events, including the amyloid accumulation discussed above [241,279]. Disruption of the desmin IFs is associated with mitochondrial mislocalization and dysfunction also in primary desmin deficiency [288,289], and this was recently shown to be normalized by CRYAB overexpression, leading to the conclusion that CRYAB has a mitoprotective function supported by a functional desmin cytoskeleton [290]. Both desmin and CRYAB interact with voltage-dependent anion channel (VDAC), which is a component of the mitochondrial permeability transition pore (PTP) and the sarcoplasmic reticulum mitochondria-associated membranes [241,290]. PTP opening and activation of the mitochondrial apoptosis pathway, which are possibly related to an increased interaction of CRYAB p.R120G with VDAC, were shown to occur in CryAB^R120G^ TG cardiomyocytes [241].

Mitochondrial dysfunction leads to increased reactive oxygen species (ROS) production, which may cause further oxidative damage to mitochondria, leading to a vicious cycle. In CryAB^R120G^ TG mice, antioxidant treatment improved mitochondrial morphology and respiratory function but did not restore mitochondrial localization [239]. Efficient normalization of mitochondrial function was achieved by TFEB transduction or intermittent fasting in hR120GCryAB mice, where stimulated autophagic flux reallowed the mitophagic removal of depolarized mitochondria [242].

Perhaps counterintuitively, a key role in the cardiac pathogenesis in hR120GCryAB mice has been demonstrated for the hyperactivation of antioxidant genes such as G6PD, leading to reducing stress (i.e., imbalance of reduced and oxidized forms of glutathione and NADPH) [237,291]. The process is thought to initially result from increased ROS production; then, it is sustained by the sequestration of Keap1, which is a negative regulator of the antioxidant response, into mutant CRYAB aggregates [237,291]. The reducing stress promotes protein aggregation and is linked to altered gene expression and perturbations in the cytoplasmic thioredoxin system on multiple levels [237,292,293].

##### Conclusions

As discussed above, dominant mutations in CRYAB can cause a multitude of defects in the target tissues. The pathogenesis of classical αB-crystallinopathy is likely to depend on a combination of gain-of-function and loss-of-function mechanisms, whose most significant ultimate cause is the amyloid aggregation of mutant CRYAB [242,258,271]. Although most of the mechanisms have been studied in the heart, the same are likely to be relevant in skeletal muscle.

In line with the central role of CRYAB aggregation in the pathogenesis, mutations leading to dominant skeletal and/or cardiac muscle disease affect either the ACD, which is predicted to disrupt the dimer interface, or the CTD (Table 5). The mutations truncating the CTD have been proposed to expose the client-binding site, increasing chaperone function toward some clients and at the same time promoting CRYAB aggregation [258]. Similarly, the C-terminal missense mutations have been suggested to affect CTD–ACD interactions [261]. For some mutations, such as p.G154S and p.*176Wext*19, further functional studies would be welcome, as they could give insight into the more atypical tissue manifestations.

##### Recessive αB-Crystallinopathy

The similar clinical and pathological phenotypes of the severe recessive infantile αB-crystallinopathy cases strongly suggest for the three reported mutations a shared pathomechanism [232,233,234]. As the recently described initiation codon mutation most likely prevents the protein expression, the phenotype seems to result from a total loss of CRYAB [234]. In this light, it is interesting that the two frameshift alleles (p.S21Afs*24 and p.S115Pfs*14) were reported to produce low levels of truncated proteins [232,233].

Functional studies on p.S115Pfs*14 revealed that the mutant protein is extremely insoluble, but its solubility is increased upon the coexpression of wild-type CRYAB [274]. Overexpression of the mutant protein in BHK21 cells produced aggregates, some of which contained desmin, and it also elicited a stress response [274]. Based on these findings, the pathogenic effect of p.S115Pfs*14 was suggested to depend on loss of function due to the unavailability of the aggregating mutant protein, with an additional toxic gain-of-function possibly contributing to the severity [274]. However, the similar phenotype of the c.3G > A (p.Met1?) mutation now argues against a major contribution from a gain-of-function component [234]. Despite interacting with wild-type CRYAB, the p.S115Pfs*14 protein does not seem to interfere with its function in heterozygous carriers, although late-onset dominant effects are not excluded [274].

Compared to the more benign adult-onset phenotype of the *Cryab/Hspb2* dKO mice [244], the severe infantile disease caused by these recessive mutations is interesting [234,274] and suggests that CRYAB is more crucial for developing muscle in humans than in mice.

### 3.3. HSPB1

HSPB1 (also known as Hsp27 in humans, Hsp25 in rodents) shows chaperone activity toward a wide range of clients [190,296,297]. It is the most widely expressed of the human sHSPs, with basal and stress-induced expression in several tissues [19,190,195]. HSPB1 is mostly present as polydisperse oligomers of 400–600 kDa [172,190], whose dramatic dissolution to smaller species is associated with the phosphorylation of the NTD [172,176,297,298,299], increased temperature [299], and oxidative conditions [300,301]. It interacts with CRYAB and HSPB6, and it forms hetero-oligomers in tissues coexpressing these sHSPs [17,19,180,302,303]. In muscle, HSPB1 shows a similar expression pattern and localization to CRYAB, albeit its absolute levels are lower [197,198,203].

A structural feature unique among the HSPB family is the single cysteine residue (Cys137), which under oxidative conditions links the subunits of the HSPB1 dimer through a disulfide bridge [304,305,306,307]. Oxidation of the cysteine may provide structural stability [304,306], increase client binding [307], and regulate HSPB1 oligomerization and activity [171,305]. The cysteine is crucial for the ability of HSPB1 to confer cellular protection against oxidative stress, although the molecular background is unknown [305,308].

The structure–function relationship of HSPB1 is complex [193]. Activation of the chaperone function is generally associated with the dissociation of oligomers to dimers [144,176,298], and recent research has demonstrated that the monomer is the most active chaperone, at least toward some clients [171,184,187]. Dimer dissociation is promoted by low pH [187], phosphorylation [184], and reduction of the intersubunit disulfide bridge [171]. However, sequential changes in the HSPB1 phosphorylation and oligomerization states following different stress treatments are complex, leading to the idea that the protein acts as a molecular stress sensor with multiple modes of protective action [193,300,309].

One such cytoprotective function of HSPB1 is protection against oxidative stress, which is mediated by increased glutathione levels due to the increased activity of G6PD and glutathione-reducing enzymes [300,308,310,311,312,313] and reduced iron uptake [273,308,314]. As mutating the Cys137 residue abolishes the protective function, it may act as a redox sensor or directly react with oxidative species [305,308]. HSPB1 has also been described a potent anti-apoptotic molecule: it suppresses apoptosis by regulating both the intrinsic and Fas-induced apoptosis pathways at several stages [220,309,315,316,317,318,319,320,321,322].

Protection of the cytoskeleton from stress-induced damage and modulating cytoskeletal structure and function are considered to be among the principal functions of HSPB1. Similarly to CRYAB, HSPB1 shows stress-induced cytoskeletal and myofibrillar association [197,203,323], and it has been shown to protect and modulate all major cytoskeletal systems: actin [184,323,324,325,326,327,328,329], intermediate filaments [208,210,330,331], microtubules [332], and titin [204]. In this regard, of particular relevance for muscle could be the recently characterized mechanosensitive functions: HSPB1, which is phosphorylated in response to mechanical stress, was shown to promote actin remodeling in strained regions of the actin cytoskeleton [329] and to modulate reversible unfolding upon filamin caused by mechanical stress [184]. The failure in the latter function leads to the irreversible damage of filamin, necessitating its removal by the CASA pathway [184].

#### 3.3.1. HSPB1 in Neuromuscular Disease

Mutations in *HSPB1* typically lead to hereditary peripheral neuropathy, which can manifest as dHMN (classified as dHMN IIb) or axonal CMT (CMT2F). These are considered as a continuum, in which mild sensory involvement becomes more prevalent with disease progression [333,334,335,336,337,338]. Of the approximately 30 identified *HSPB1* mutations, most have a dominant effect, although some recessive or semidominant mutations have also been described [334,337,339]. HSPB1 mutations are the most frequent cause of hereditary neuropathy [338]; in the cohort of 510 dHMN index patients studied by Echaniz-Laguna and colleagues, HSPB1 accounted for 5.5% the cases [337]. In a large CMT cohort (17,880 patients), the proportion of HSPB1 was approximately 0.5% [340]. Disease mutations are located throughout the gene; a selection of these is presented in Table 6.

Neuropathies caused by *HSPB1* mutations are usually rather benign in their clinical course. In a recent natural history study by Rossor and colleagues, the average age of onset was 40 [338], but there is considerable variation between families [337,338,341]. From initial distal lower limb weakness, manifesting as foot drop, the symptoms progress slowly in a length-dependent manner to distal upper limbs and proximal legs [337,338,341]. CNS involvement, namely pyramidal or cerebellar symptoms, is found in a small subset of patients [337].

A more dramatic, rapidly progressing ALS-like phenotype has been reported in some patients with *HSPB1* mutations [342,343]. A heterozygous variant in the heat shock element of HSPB1 promoter, interfering with basal expression and stress inducibility of the gene, was also identified in an ALS patient [344].

Recently, a vacuolar distal myopathy in combination with motor neuropathy has been described in a few patients, expanding the phenotypic range to myogenic symptoms [339,345].

#### 3.3.2. Pathomechanisms of HSPB1 Mutations

The effects of disease-causing HSPB1 mutations have been characterized in various systems, from basic biochemical studies to cell and animal models. These studies have revealed the diverse effects of the mutations on the functions and properties of HSPB1 (Table 6). As the studies have utilized different methodologies and most have included only a few variants, a detailed comparison of the mutations is not feasible. However, some unifying themes emerge from the findings.

##### Properties of Mutant Proteins 

Several pathogenic missense mutations are clustered at the β5 and β6+7 strands of the ACD and the intervening loop [171]. Many of these variants affect the dimer interface [170,171,346,347], and some have been shown to affect the homodimerization of HSPB1 [170] or its heterodimerization with HSPB6 [346]. In line with the role of the dimer interface in client binding, the reduced dimerization of p.R127W and p.S135F was found to be accompanied with potentiated chaperone function toward some clients and increased binding to client proteins, and it was even reflected in improved cells survival after heat shock [170,347]. Of the ACD mutations not located in the dimer interface, most reside in other regions showing dynamic structure in the active HSPB1 monomer, underlining the important role of these regions for HSPB1 function [171].

The p.R140G mutation, although located at the dimer interface, does not disrupt ACD dimerization and was found to significantly decrease the chaperone activity of HSPB1 in vitro [346]. This mutation corresponds to the pathogenic p.R120G change in CRYAB (see above), and it could therefore have similar molecular consequences. Indeed, the p.R140G mutation impairs the ability of HSPB1 to inhibit desmin gel formation, although the defect is less severe than that seen with CRYAB p.R120G [210]. Both mutant proteins show increased oligomer size and aggregation propensity [262,294,346]; however, amyloid formation by HSPB1 p.R140G has not been reported. As the p.R120G mutation interferes with the dissociation of CRYAB dimers in low pH [179,254,255], the same could be true for HSPB1 p.R140G and possibly other ACD mutations involving basic residues [187].

Interestingly, the C-terminal extension p.A204Gfs*6, which is associated with an ALS phenotype, was found to cause impaired dimer dissociation as well: this mutant protein sequesters wild-type HSPB1 to stable heterodimers with impaired chaperone function [343]. Of the other HSPB1 mutations outside the ACD, some have been demonstrated to decrease the chaperone activity in in vitro assays, whereas others have shown little or no effect (Table 6) [343,348,349,350].

Biophysical and biochemical characterization has shown that many mutations affect HSPB1 oligomerization, which may contribute to the dysregulation of chaperone activity and/or other functions of HSPB1. In size exclusion chromatography, analytical ultracentrifugation, and dynamic light scattering experiments, several of the mutant proteins show enhanced heat-dependent self-association and form larger oligomers [346,347,348,349,350]. However, the mutant oligomers differ in their dynamics. Some dissociate more readily than the wild-type protein [346,347,348,349] and show increased sensitivity to phosphorylation, causing the same level of phosphorylation to lead to a more pronounced dissociation of the oligomer [347,348]. Others, in particular mutations affecting the NTD, show impaired oligomer dissociation and attenuated response to phosphorylation [350]. The altered oligomerization properties of some NTD mutants were recently explained on the structural level by their altered interactions with the ACD [138].

The substitutions affecting Pro182 in the CTD stand out due to their effect on HSPB1 solubility. The mutant proteins form aggregates that may also recruit wild-type HSPB1 or client proteins [170,349,351]. The mutated residue is part of the IPI/V motif that mediates the interaction of the CTD with the ACD, and mutations presumably lead to increased interdomain interactions within or between HSPB1 monomers [349].

##### Downstream Pathomechanisms

The dominant pathogenic effects of HSPB1 mutations have been demonstrated in vivo in several mouse models. Mice with neuronal overexpression of human HSPB1 p.S135F or p.P182L developed axonal motor neuropathy and muscle denervation, which is evident as progressively impaired motor performance and a decrease in muscle strength [352]. While the motor phenotype was more severe in the p.P182L model, the p.S135F mouse showed also sensory neuropathy, consistently with the effects of these mutations in human patients [352]. The findings were corroborated by another model overexpressing HSPB1 p.S135F, which, in addition to axonal neuropathy, showed also demyelination [353]. Neuropathological and electrophysiological changes were also found in the mouse overexpressing HSPB1 p.R136W in neurons, although this model did not develop a clinical phenotype [354]. In contrast to the overexpression models, mice expressing the p.S135F or p.P182L mutant proteins at a more physiological level did not develop even subclinical neuropathy [355].

There is substantial evidence that some of the pathogenic effects of HSPB1 mutations are mediated by cytoskeletal abnormalities affecting the neurofilament (NF) and microtubule (MT) networks. A few mutations have been shown to disrupt NF assembly in vitro [333,351,356], and NF alterations have also been observed in mouse models expressing HSPB1 mutations [353,354]. These changes may depend on the aggregation of NF proteins with mutant HSPB1 [333,351,356] or their reduced axonal transport, which correlates with increased NF phosphorylation by Cdk5 [357]. Furthermore, in vitro studies of Nefedova et al. hinted for several HSPB1 mutants an increased interaction with NFs, although the differences did not reach statistical significance [331]. A direct role for NFs in the pathogenesis was indicated by Zhai and colleagues, who showed that neurotoxicity of HSPB1 p.S135F for cultured motoneurons was diminished in NFL-deficient cells [356].

HSPB1 plays a role in the formation of non-centrosomal MTs [332], and mutations at the dimer interface have been shown to increase its binding to tubulin and MTs and disturb MT dynamics in transfected cells and primary neurons by overstabilizing MTs [358]. Decreased tubulin acetylation is a feature of mouse models expressing mutant HSPB1 [352,353,359], and this was suggested to reflect a secondary change in response to MT hyperstability [358].

Cytoskeletal defects, tubulin hypoacetylation in particular, may lead to neurodegeneration by disturbing the axonal transport of proteins and organelles. Indeed, a decreased proportion of moving mitochondria and/or lower mitochondrial velocity have been reported in neurons derived from the HSPB1 p.S135F mouse model [352] or from patient-derived induced pluripotent stem cells (iPSCs) [359] or transduced with mutant proteins [360]. The defects are relevant for the pathogenesis and seem to be at least partly due to MT hypoacetylation, as HDAC6 inhibitors can correct the mitochondrial transport in vitro [352,359,361] and reverse the neuropathic phenotype of the HSPB1 p.S135F mouse model in vivo [352].

The role of mitochondrial defects in the downstream pathomehcanism of HSPB1 mutations gained additional support from recent studies [360,362]. In the experiments of Kalmar and colleagues on cultured motor neurons, the expression of HSPB1 mutants, most remarkably p.S135F, decreased mitochondrial membrane potential in neurites and impaired complex I activity [360]. This was accompanied with decreased glutathione levels and increased oxidative stress [360]. As the retrograde transport of p75NTD was affected to a lower extent, a primary impairment in mitochondrial function, rather than a cytoskeletal defect, was suggested to underlie the altered mitochondrial transport in this model [360]. One mechanism for this was identified by Schwartz et al., who showed that an abnormal interaction of mutant HSPB1 with ceramide synthase led to decreased mitochondrial ceramide content and impaired respiration [362].

The lack of an overt phenotype in HSPB1-deficient mice [363] suggests that the pathomechanisms of dominant HSPB1 mutations involves a gain-of-function component [352]. However, the loss of cytoprotection is another potential consequence of HSPB1 mutations, which may contribute to the pathomechanism in combination with dominant toxicity [364]. Impaired tolerance to thermal and unfolded protein stress has been observed in patient fibroblasts [365]. HSPB1 was shown to protect cells from ER-stress-induced apoptosis by facilitating the proteasomal turnover of BIM, and several disease mutants are defective in this respect [322]. Recent research has demonstrated as well that non-cell autonomous protective effects may be altered by the mutations: while wild-type HSPB1 expressed in astrocytes with SOD1 p.G93A protects co-cultured motoneurons from mutant SOD1 toxicity, the p.G84R and p.R136W fail to show such a protective effect [364].

The p.P182L mutation was recently shown to increase the interaction of HSPB1 to the RNA-binding protein PCBP1, interfering with its ability to suppress the translation of its target genes—some which are known neuropathy genes [366]. HSPB1 p.R127W did not show the same effects, suggesting a mechanism specific for p.P182L and possibly other C-terminal mutations [366].

Very recently, autophagy was added to the list of mechanisms potentially affected by HSPB1 mutations. HSPB1 was shown to interact with SQSTM1/p62 and promote macroautophagy by driving the formation of SQSTM1 bodies and phagophores [367]. HSPB1 mutants (p.R127W, p.S135F, and p.P182L) showed stronger interaction with SQSTM1 and impaired autophagic flux [367]. Decreased autophagic flux was also seen in motor neurons derived from patient iPSCs, supporting the relevance of this mechanism for the pathogenesis [367].

In summary, disease-causing mutations exert pleiotropic deleterious effects on the HSPB1 protein, and the pathogenesis of CMT/dHMN is likely to depend on a combination of downstream mechanisms that may be different for different mutations. Interestingly, a structural connection between the two neuromyopathy-associated *HSPB1* mutations suggests that the myopathy phenotype may involve a distinct pathomechanism, which is possibly analogous to α-crystallinopathy [345]. The p.D129E mutation affects the Asp residue predicted to form a salt bridge with Arg140 at the dimer interface, similarly to the Asp109–Arg120 pair in CRYAB [345]. Therefore, Lewis-Smith and colleagues predicted that mutations in Arg140 should also cause myopathy [345] and, indeed, p.R140G in the homozygous state was identified in a patient with a neuromyopathic phenotype [339].

### 3.4. HSPB3

HSPB3 is, at 17 kDa, the smallest member of human HSPBs [19,371]. It shows by far the highest expression in heart, followed by skeletal muscle and, accordingly, its expression is induced in vitro in myotube differentiation [18,19]. In addition, HSPB3 has been detected in smooth muscle and different fetal tissues [371], regionally in the CNS [372,373,374], and both in sensory and motor neurons in the peripheral nervous system [375].

#### 3.4.1. Functions of HSPB3

In contrast to other sHSPs, HSPB3 does not seem to homodimerize [19]. Instead, HSPB3 associates with HSPB2, which is another sHSP prominently expressed in cardiac and skeletal muscles [19,376]. The proteins form heterotetramers with a HSPB2:HSPB3 subunit ratio of 3:1, and these assemble into distinct oligomers [377,378]. As the majority of HSPB3, at least in striated muscles, is associated with HSPB2 [19,377], the functions of the two chaperones need to be considered together.

In muscle, HSPB2 has been detected at or near the Z-discs, in cardiac intercalated discs, and in neuromuscular junctions [197,376,379]. While HSPB2 and HSPB3 are not heat-inducible in contrast to canonical sHSPs [19], a role in proteotoxic stress management is supported by their upregulation in the SBMA mouse model expressing a polyQ-expanded androgen receptor [380]. In different experimental setups, HSPB2 and HSPB3 alone or in complex have shown varying degrees of client-dependent chaperone activity in vitro or in cultured cells [376,377,381,382,383,384], and overexpressed HSPB2 also improved the thermotolerance of transfected cells [385].

The association of HSPB2 with mitochondria in myotubes [385] and interaction with a large number of mitochondrial proteins [384] have suggested that HSPB2 has a role in protecting mitochondria from stress. Indeed, the cardiac overexpression of HSPB2 was shown to confer mitochondrial protection in an ischemia/reperfusion model [384], whereas HSPB2 deficient-mice showed altered mitochondrial function in cardiac overload [386]. On the other hand, the stress-induced association of HSPB2 and/or HSPB3 with sarcomeric and cytoskeletal structures suggests a role in the stabilization of these proteins [197,376,387,388]. This notion is supported by the interactions of HSPB2 with several myofibrillar and cytoskeletal components, including actin, myosin, titin, and filamin [384].

The role of HSPB2/HSPB3 in developing muscle was recently elucidated by Morelli and colleagues, who showed that the intrinsically disordered C-terminal region of HSPB2 mediates its compartmentalization into droplet-like membraneless organelles through liquid–liquid phase separation [389]. Overexpressed HSPB2 was found to form nuclear compartments that altered the distribution of lamin A and chromatin and suppressed transcription, and this could be prevented by HSPB3 coexpression [389]. In differentiating myotubes, endogenous HSPB2 was shown to form both cytosolic and nuclear foci, and the authors proposed that this compartmentalization mediates the nuclear remodeling known to take place during muscle differentiation [390]. The important role of HSPB3 in this context appears to be the negative regulation of HSPB2, preventing its inappropriate phase separation [389]. Interestingly, the absence of HSPB3 from nuclear HSPB2 foci [389] suggests that a yet unidentified factor may regulate the phase separation of HSPB2 by modulating its interaction with HSPB3. Another interesting question is whether the functions of HSPB2/HSPB3 in mature muscle depend on the phase separation of HSPB2.

Given the causative role of HSPB3 mutations in neuropathies (see below), its expression and functions in the nervous system are of interest. In peripheral neurons, HSPB3 showed, in addition to cytosolic localization, association to the actin and neurofilament cytoskeleton, and mitochondria [375]. A functional role in neuron survival was supported by *in ovo* studies on chicken embryos, where singly overexpressed HSPB3 decreased the number of motor neurons, but it showed a neuroprotective effect in the limb bud removal assay [375].

It is not known if the functions of HSPB3 outside striated muscles are related to its complex with HSPB2. As the expressions of HSPB3 and HSPB2 do not always correlate [19,373], it seems plausible that HSPB3 could in some situations complex with other partners; however, no definitive evidence exists for binary interactions with other sHSPs [19,391]. La Padula et al. speculated that HSPB3 could in neurons associate with HSPB8 [375]. While yeast two-hybrid studies did suggest binding of HSPB3 to HSPB8, this was not supported by FRET [391].

Another unclear issue is whether HSPB2 and HSPB3 can engage into physiologically relevant interactions with BAG3. In vitro, singly expressed HSPB2 shows weak binding to BAG3, but this is disrupted in the presence of HSPB3 [18]. HSPB8, in turn, interacts with the binding site in BAG3 with higher affinity than HSPB2 [18]. Consequently, in myotubes, where on one hand highly expressed HSPB8 competes with HSPB2 for BAG3 binding, and on the other hand HSPB3 competes with BAG3 for HSPB2, the only complex present in detectable amounts was that of HSPB8 with BAG3 [18]. However, the competing interactions could in some conditions produce alternative complexes [18].

#### 3.4.2. HSPB3 in Neuromuscular Disease

Dominant mutations in HSPB3 are a rare cause of neuropathy and myopathy: since the identification of first pathogenic *HSPB3* mutation by Kolb and colleagues 10 years ago [392], only a few additional ones have been published (Table 7) [389,393]. The missense changes p.R7S and p.Y118H have been reported in single families with dominantly inherited axonal neuropathy, the clinical phenotype ranging from motor-predominant with mild sensory involvement to a sensorimotor CMT phenotype [392,393]. Two additional dominant HSPB3 mutations, p.L34Ffs*50 and p.R116P, were reported in patients with myopathy [389]. The patient with the frameshift mutation was described as having shoulder-girdle weakness and atrophy, whereas the p.R116P missense patient presented with muscle weakness and axonal neuropathy, and in EM showed myofibrillar disarray and a range of other abnormalities, including deformed nuclei [389]. At this stage, the small number of cases and the limited detail of the published clinical descriptions [389,392,393] do not allow determining if the “neuropathy” and “myopathy” patients represent distinct diseases or a phenotypic continuum, nor evaluating possible genotype–phenotype correlations.

The HSPB3 mutations apparently cause disease through distinct pathomechanisms. The missense mutations p.R116P and p.Y118H affect closely spaced residues at the ACD β6+7 strand, which is located at the HSPB2/HSPB3 heterodimer interface [378,393]. Of note, the former involves the highly conserved basic residue homologous to the mutation hotspot in several sHSPs (HSPB1, CRYAB and HSPB8). Morelli and colleagues demonstrated that p.R116P causes the aggregation of HSPB3 as well as abolishes its interaction with HSPB2, thereby preventing the ability of HSPB3 to regulate HSPB2 phase separation [389]. In an experimental setup, the inappropriate phase separation of HSPB2, which was not inhibited by mutant HSPB3, led to the formation of excessive HSPB2 compartments in the cytosol and nucleus, sequestering lamin A to the nuclear foci and interfering with gene expression [389].

The frameshift allele produces an abnormal protein that is subject to rapid degradation by the proteasome and hence is unable to regulate HSPB2 [389]. Hence, this mutation seems to share the downstream pathomechanism with p.R116P and probably p.Y118H. The nuclear abnormalities observed in p.R116P patient muscle are in line with the idea that the inappropriate nuclear phase separation of HSPB2 plays a pathogenic role in the human patients [389]. It is of note that the dominant effect of the mutations suggests that the regulation of HSPB2 is highly sensitive to the correct levels of functional HSPB3. In addition, as pointed out by the authors, a toxic gain-of-function related to HSPB3 aggregation may also contribute to the pathomechanism of the p.R116P mutation [389].

In contrast to the other reported mutations, p.R7S does not disrupt the interaction with HSPB2 [389]. The R7 residue is located in HSPB3 NTD, which is close to the IXI/V motifs thought to mediate oligomerization, and the mutation was shown to have a relatively mild effect on higher-order HSPB2/HSPB3 oligomer formation [378]. The functional consequences of this alteration remain to be been characterized. In the *in ovo* experimentation by La Padula et al., some differences in neuron survival were seen between wild-type and p.R7S, although these did not reach statistical significance [375].

### 3.5. HSPB8

HSPB8 (Hsp22) shows widespread expression, with the clearly highest levels observed in cardiac and skeletal muscles [394,395,396], and it is induced in proteotoxic stress [397,398,399,400,401,402]. Unlike most other sHSPs, HSPB8 does not seem to form homo-oligomers nor hetero-oligomers with other sHSPs: HSPB8 homodimers form larger oligomers only at high concentrations [189,190], and while interactions of HSPB8 with several other sHSPs have been detected in experimental conditions [20,391,394,403], these are relatively weak [404,405].

An important functional partner of HSPB8 is BAG3, which associates with an HSPB8 dimer to a 2:1 complex [15]. Although BAG3 is able to bind multiple different sHSPs, the special relationship of HSPB8 and BAG3 is illustrated by the high affinity of the two proteins and the decreased stability of HSPB8 in the absence of BAG3 [15,16,406]. Therefore, BAG3 has been considered an obligate partner for HSPB8. However, interestingly, recent research has demonstrated that some of the functions of HSPB8 are actually independent of BAG3 [407,408,409]. Hence, it is not clear to what extent the reported functions and effects of HSPB8 depend on BAG3-dependent and independent pathways.

HSPB8 shows phosphorylation-regulated chaperone activity in vitro [169,189,190,262,381,410,411,412,413]. While HSPB8 was the most effective of the chaperones tested by Bruinsma et al. in preventing α-synuclein fibrillization [381], purified HSPB8 is generally not a particularly effective chaperone in vitro, suggesting specificity for certain clients [190]. However, in the cellular context, HSPB8 has been shown to efficiently promote the autophagic clearance of misfolded and/or aggregation-prone proteins, including many disease-associated mutant proteins such as mutant SOD1, polyQ-expanded androgen receptor and huntingtin, TDP-43, and C9ORF72-derived dipeptide repeat proteins [15,397,400,409,411,414,415,416,417,418,419].

The function of HSPB8 in promoting autophagy depends on its interaction with BAG3. Autophagic degradation of ubiquitinated client proteins through the CASA pathway (discussed in more detail below) is directly mediated by the HSPB8–BAG3 complex, to which HSPB8 is thought to provide client-binding capacity [15,420,421]. In addition, HSPB8 and BAG3 may also stimulate pro-autophagic signaling and suppress protein translation by promoting eIF2α phosphorylation, which is a pathway demonstrated to contribute to their effect against polyQ aggregates [422].

Upstream of autophagy, HSPB8 promotes the early sequestration of client proteins to ubiquitinated microaggregates in a BAG3-independent manner, and through interactions with BAG3 and SQSTM1, it promotes the transport of clients to the aggresome [408]. Notably, the common functions of HSPB8 and BAG3 are not limited to sequestration and degradation: at least in vitro, the two proteins act together to promote the refolding activity of HSPA [16].

Recent work by Li et al. indicated for HSPB8 a function also in mitophagy [423], and this was suggested by the authors to contribute to the protective effects of HSPB8 in ischemia–reperfusion and oxidative stress models [424,425,426,427]. BAG3 has been shown to promote mitophagy as well [428], and it is not known whether the two proteins act together in this function. With regard to mitochondria, analysis of cardiac-specific HSPB8-deficient and overexpressing mice has shown that HSPB8 has a stimulatory effect on respiration, which is mediated by effects on STAT3-dependent stress signaling and the HSPB8-dependent mitochondrial localization of iNOS [429,430]. These changes in the mitochondrial function are considered to underlie the increased susceptibility of HSPB8-deficient mice to heart failure after pressure overload and, on the other hand, hypertrophy and increased oxidative stress in the overexpressing model [429,431]. In this context, it should be mentioned that *Drosophila* Hsp22—a constitutively mitochondrial sHSP in the fly—is not the closest functional ortholog of HSPB8 [432,433], and the mitochondrial functions of the two need not necessarily be the same.

In addition to autophagy, HSPB8 may also play a role in proteasomal degradation. This was suggested by the increased proteasome expression and activity in the cardiac HSPB8-overexpressing mouse model and the physical interaction of HSPB8 with the proteasome [434].

#### 3.5.1. HSPB8 in Neuromuscular Disease

Mutations in *HSPB8* typically lead to a continuum of hereditary neuropathies ranging from dHMN (type IIa) to CMT (type 2L), which are highly variable in the clinical course [337,435,436,437]. The clinical phenotypes are very similar to the neuropathies caused by *HSPB1* mutations, as discussed above [336,337]. Remarkably, the disease mutations (Table 8) most often affect the single hotspot residue Lys141 (corresponding to Arg140 of HSPB1 and Arg120 of CRYAB; see above): mutations leading to four different missense changes of this residue (p.K141N, p.K141E, p.K141T and p.K141M) have been so far identified [337,435,436,437]. Two missense mutations affecting other residues, p.P90L and p.N138T, were described in dHMN patients only recently [337].

Apart from the typical pure neuropathy phenotype, *HSPB8* mutations may cause myopathy, with or without neurogenic involvement [438,439,440,441]. Ghaoui and colleagues first identified the heterozygous mutations c.421A>G (p.K141E) and c.515dupC (p.P173Sfs*43) in patients featuring motor neuropathy in combination with distal-onset myopathy [438]. Both of these variants have been later found in additional families [440,441]. Another frameshift mutation c.508_509delCA (p.Q170Gfs*45), resembling the previously reported one on the protein level, was identified by Echaniz-Laguna and colleagues in several unrelated families with axial and distal myopathy [439]. These patients showed muscle involvement similar to that reported for p.P173Sfs*43 [438] with additional involvement of paraspinal muscles, but there was no associated neuropathy [439]. The reported pathological features in all HSPB8-related myopathies have been similar: dystrophic changes and MFM pathology with protein aggregates (desmin, myotilin, CRYAB, dystrophin, HSPB8, DNAJB6, myotilin, BAG3, TDP-43, and ubiquitin) and rimmed vacuoles [438,439,440,441].

#### 3.5.2. Animal Models

Different mouse models have been established to study the effects of HSPB8 deficiency and mutations in vivo [396,429,442,443]. The most informative of these are the knock-in (KI) model recently described by Bouhy and colleagues, and the knockout (KO) model created in parallel [443].

The KI model, with the p.K141N mutation introduced in murine HSPB8, recapitulates well the effects of HSPB8 mutations in human patients [443]. Homozygotes develop a motor neuropathy with progressive axonal degeneration. The neuropathology, with the accumulation of mitochondria and other degenerating organelles, is suggestive of impaired axoplasmic flux [443]. On the physiological level, the disease manifests as declining strength and locomotor performance, which is apparent from 9 months of age [443]. The electrophysiological and neuropathological changes and locomotor deficit are similar to those reported by Zhang et al. in a transgenic mouse model ubiquitously overexpressing human HSPB8 p.K141N [442].

In addition to the neuropathy, homozygous p.K141N KI mice develop a myopathy phenotype, which is presumably of myogenic origin and thus independent of the motor neuron defect [443]. In line with human patients with HSPB8-related myopathy, the muscle pathology shows features of human myofibrillar myopathy, with Z-disc disintegration, accumulation of granulofilamentous material, aggregates positive for HSPB8, CRYAB, and desmin, and rimmed vacuoles [443].

Although heterozygous p.K141N KI animals showed normal performance in functional tests, ultrastructural analysis of nerves and muscles revealed similar pathological changes as seen in homozygotes [443]. Importantly, HSPB8 KO animals did not develop a motor phenotype nor myofibrillar myopathology, indicating that the main phenotypic features seen in the homozygous KI mice were due to a dominant toxic effect of the HSPB8 p.K141N rather than loss of function [443].

Very interestingly, the two reported *Hspb8* KO models have normal lifespan with no overt disease phenotype [429,443]. However, Bouhy et al. observed an accumulation of abnormal mitochondria in the muscles of their KO animals [443], and this is in line with the functional alterations in cardiac mitochondria reported by Qiu et al. [429].

Recently, Jabłońska and coworkers developed *Drosophila* models based on the muscle-specific overexpression of fluorescently tagged Hsp67Bc, which is the fly ortholog for HSPB8 [433,444]. In this context, the mutations p.R126E and p.R126N (equivalent to HSPB8 p.K141E and p.K141N) had different phenotypic outcomes [444]. The p.R126E mutant flies showed changes such as myofibrillar disorganization, altered neuromuscular junctions, and mitochondrial disruption and depolarization, which were reflected in impaired muscle function [444]. On the other hand, the p.R126N mutation caused a massive aggregation of the mutant protein but less severe sarcomeric alterations and no signs of mitochondrial abnormalities [444]. In functional assays, these flies showed normal muscle performance [444].

#### 3.5.3. Pathomechanisms of HSPB8 Mutations

Functional studies, mostly done with p.K141E and p.K141N, have demonstrated for HSPB8 mutations a combination of gain-of-function and loss-of-function effects (Table 8), with many similarities to HSPB1 and CRYAB mutations.

##### Chaperone Activity and Autophagy

In vitro work utilizing purified proteins demonstrated that the chaperone activity of HSPB8 p.K141E is, depending on the client, normal or clearly impaired [189,413]. While these studies have assayed the holdase function with specific clients, cell-culture-based experiments have offered a more comprehensive view on the effects of mutations—the flipside being that the specific pathway(s) responsible for the defects are more difficult to pinpoint. Using different aggregation-prone clients (polyQ proteins, mutant SOD1, HSPB1, p.P182L), such studies have shown that the disease mutations impair the ability of HSPB8 to prevent the formation of aggregates and/or promote their clearance [397,411,433,438,445]. These effects may partially be due to compromised client binding and partially to defective autophagy. Indeed, Kwok et al. showed that while wild-type HSPB8 stimulates autophagy in NSC34 cells, the p.K141N mutant suppresses it compared to the baseline situation, which is due to blocked autophagic flux at the level of autophagosome–lysosome delivery or fusion [445].

##### Aggregation and Cytotoxicity

When expressed in cultured cells, mutant HSPB8 constructs have been reported to exhibit varying degrees of cytotoxicity [337,396,435,446,447]. Irobi and colleagues reported cytotoxic effects of p.K141N and p.K141E in the N2a neuronal cell line [435] and in primary motoneurons, where transduced cells showed neurite degeneration [446]. Less pronounced neurite degeneration was seen in primary sensory neurons expressing the p.K141E mutant protein, whereas primary cortical neurons or glial cells showed no signs of cytotoxicity [446], suggesting that motor neurons are most susceptible to the toxic effects of mutant HSPB8 [446]. For the more recently described mutations, mild cytotoxicity has been reported in cardiomyocytes, as well as SH-SY5Y and N1E-115 neuroblastoma cells [337,396]. Neurotoxic effects are supported by the decreased number and morphological abnormalities of sensory neurons in patient skin biopsies [448].

The cytotoxicity of mutant HSPB8 has been suggested to depend on its aggregation propensity, which was observed for p.K141E and p.K141N in several in cell models [20,396,435,447,448], including primary patient fibroblasts [448], as well as in vivo in HSPB8 p.K141N-expressing mouse models [396,443]. However, the relationship of aggregation with cytotoxicity is not entirely clear. The cytotoxic effects seen for various mutant constructs in SH-SY5Y cells [337] and primary motor neurons [446] were not associated with microscopically detectable aggregation. Similarly, in *Drosophila* models, the visibly aggregating Hsp67Bc p.R126N protein was associated with a less severe phenotype than p.R126E [444]. These observations could be in line with the idea that the most cytotoxic species is a soluble preamyloid oligomer, which is thought to be the case with CRYAB [236,277]. Indeed, Sanbe and colleagues demonstrated that recombinant HSPB8 p.K141N forms amyloid oligomers in vitro similarly to CRYAB and shows cytotoxic effects when added to cell culture media [396]. However, the mild effects of HSPB8 compared to CRYAB mutations led the authors to suggest that the amyloidogenic properties are not fully correlated with cytotoxicity [396].

Aggregates of mutant HSPB8 may also recruit other proteins of the PQC machinery, as demonstrated with HSPB1 and HSPA [435,448]. Along these lines, the p.K141N and p.K141E mutations have been found to increase the binding of HSPB8 with HSPB1, CRYAB, and HSPB8 self-association [20,435]. Regarding the HSPB8–BAG3 interaction, the picture is unclear: while some mutations have shown no effect on BAG3 binding, others have indicated decreased or increased binding with partly contradictory results [15,183,337,433].

##### Mitochondria and Oxidative Stress

Several lines of evidence suggest that one of the downstream pathways mediating the pathogenic effects of HSPB8 mutations is mitochondrial dysfunction, which can be due to loss of protective or stimulatory functions or toxicity of the mutant proteins, or perhaps both. In addition to the fly models discussed above, alterations in mitochondrial function have been observed in patient fibroblasts [448], SH-SY5Y cells transfected with HSPB8 p.K141N [447], as well as in hearts of p.K141N-overexpressing mice [396]. Sanbe and colleagues also noted an increased mitochondrial localization of HSPB8 p.K141N in cardiomyocytes and transgenic hearts, and—similarly to CRYAB p.R120G—increased interaction with VDAC, although the functional significance of these changes is unclear [396]. On the other hand, the ability of recombinant HSPB8 p.K141N to suppress the oxidative phosphorylation of isolated mitochondria clearly speaks for direct toxicity of mutant HSPB8 [396].

In SH-SY5Y cells expressing HSPB8 p.K141N, the aggregation of mitochondria and the mutant HSPB8 was accompanied by mitochondrial depolarization, increased ROS levels, and reduced cell viability [447]. Interestingly, HSPB8 p.K141N expression in this model was associated with a reduced nuclear level of NRF2, which is the transcription factor driving antioxidant response. This suggests that the mutation may—as recently suggested for the BAG3 p.P209L mutation—interfere with the ability of HSPB8–BAG3 to regulate the p62–KEAP1–NRF2 pathway [408,447]. Notably, NRF2 localization, mitochondrial parameters, and cell viability were reversed by the antioxidant L-3-n-butylphthalide, which is a treatment that also increased the neurite number in motor neurons transduced with HSPB8 p.K141N, further supporting the relevance of this pathway in the pathomechanism of HSPB8 mutations [447].

##### RNA Metabolism

HSPB8 mutations may also have downstream effects on RNA metabolism. This is suggested by the altered binding of mutant HSPB8 (p.K141N and p.K141E) to the RNA helicase DDX20 [449]. This component of the SMN (survival of motor neuron) complex and snRNPs is involved in transcriptional regulation and RNA processing, with functional connections to proteins associated with motor neuron degeneration, such as TDP-43 [450,451]. Along the same lines, decreased TDP-43 expression and altered splicing of TDP-43 target genes was recently reported in a muscle samples from patient with HSPB8-related neuromyopathy [440].

##### HSPB8-Related Myopathy

The two frameshift mutations, p.Q170Gfs*45 and p.P173Sfs*43, appear to preferentially affect muscle, as they have been so far only reported in (neuro)myopathy patients [438,439,441]. With the 24–27 C-terminal amino acids of HSPB8 replaced by 43–45 erroneous residues, the protein products of these mutant alleles are nearly identical, and they very likely act through a shared pathomechanism, which could be partially distinct from the missense mutations. These mutations are associated with a 40–60% decrease of HSPB8 protein in patient muscles and fibroblast cultures, with no sign of expression of the extended species [439,441]. As nonsense-mediated mRNA decay should not be triggered by these mutations located in the last exon of *HSPB8*, the data suggest rapid degradation of the mutant proteins [439,441].

Based on the decreased HSPB8 expression, the pathogenicity of p.Q170Gfs*45 was suggested by Echaniz-Laguna and colleagues to depend on haploinsufficiency [439]. However, in light of the KI and KO mouse models discussed above [429,443], a gain-of-function is perhaps a more likely explanation, notwithstanding the apparent absence of the mutant protein. This enigma might be related to the findings of Al-Tahan et al., whose immunofluorescence analyses revealed increased HSPB8 protein levels in p.P173Sfs*43 patient fibroblasts after heat shock [441]. While this could simply reflect slower clearance of HSPB8-decorated aggregates due to impaired autophagic flux, it could indicate accumulation of the mutant protein itself.

Given the role of HSPB8 in the CASA pathway, which is reported to be essential for muscle maintenance [421], HSPB8-related myopathy has been proposed to be due to impaired CASA function [438,441]. This connection to CASA is supported by the myofibrillar/rimmed-vacuolar changes that resemble the pathology caused by BAG3 mutations [438,439]. Moreover, LC3B and SQSTM1 accumulation in patient fibroblasts suggests impaired autophagic flux [441]. However, the benign of phenotypes of *Hspb8* KO mice [429,443] again indicate that a simple loss of CASA does not result in MFM pathology, or that other sHSPs may compensate for the lack of HSPB8, at least in mice.

Whereas a muscle-specific pathomechanism can be envisioned for the frameshift mutations, the myopathy phenotype caused by HSPB8 p.K141E, which is typically associated with neuropathy, is more intriguing [438,440]. The muscle involvement in these patients could be determined by genetic modifiers, as exemplified by the recently demonstrated digenic effect of TIA1 and SQSTM1 [452], or environmental factors. Moreover, as pointed out by Ghaoui et al., the considerable involvement of proximal muscles sometimes seen in HSPB8-related neuropathy patients could be explained by an undetected myopathy component [438].

## 4. BAG3

The cochaperone BAG3 (Bcl-2-associated athanogene 3, or BAG family molecular chaperone regulator 3) is a member of the BAG-protein family, which is defined by the presence of at least one BAG domain. In humans, there are six *BAG* genes: *BAG1*–*BAG6* [158,453,454,455]. Of these, BAG1–BAG5 contain canonical BAG domains, whereas BAG6 has a BAG-like domain with a separate function [456]. Still, BAG6 has been shown to interact with HSPA in coimmunoprecipitation experiments [457] and is by most considered a BAG-family protein. Apart from the common BAG-domain, the BAG proteins are structurally very different and have distinct cellular functions.

All canonical BAG proteins interact directly with the ATPase domain of HSPA chaperones through the BAG domain. Upon binding, the BAG protein acts as a NEF and stimulates the release of bound ADP from HSPA [158]. In turn, this causes the release of the client protein from the client-binding domain of HSPA [458], thereby preparing HSPA for a new cycle of chaperonal activity. Apart from the interaction of the BAG domain with ATPase domain, other parts of BAG3 have been found to interact with the client-binding domain of HSPA, promoting client release in vitro [459].

Each of the BAG proteins has different affinity for HSPA; this was studied in detail for HSPA1A, which was found to bind BAG3 with higher affinity than the other BAG proteins [458]. Using the combined expression of various JDPs and BAG proteins, it was also shown that the in vitro ATPase activity of HSPA1A depends on JDP identity and the stoichiometry of JDP versus BAG [458]. The same has also been shown for BAG3 and HSPB8, where a clear stoichiometric optimum exists for optimal HSPA activity [16]. This clearly shows the importance of the cellular background for functional studies, and it could be one reason for functional assays giving different results in different experimental setups and cell lines, as well as explain the tissue specificity of certain diseases.

For further information of the BAG family members, we refer to a recent review by Behl [453] and references therein, and we will focus on the only BAG protein where disease-causing mutations have been identified: BAG3.

### 4.1. Structure and Functions of BAG3

BAG3 is a protein of 575 amino acids, with a theoretical molecular weight of 61.6 kDa, but it consistently migrates at a higher weight in Western blots—normally at approximately 75 kDa. BAG3 is highly conserved and is expressed in all mammalian tissues, with highest expression in cardiac and skeletal muscle, but also in many cancer tissues [108,460,461,462,463]. In skeletal muscle, BAG3 primarily localizes to the Z-disc and the sarcolemma [108,464].

**Figure 6:**
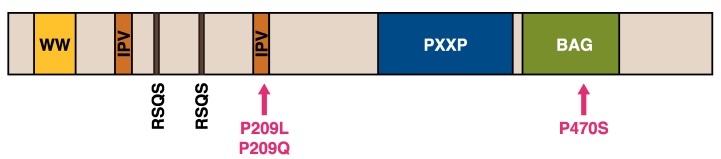
Schematic structure of BAG3 with domains and motifs, and myopathy-causing mutations indicated. BAG3: Bcl-2-associated athanogene 3, or BAG family molecular chaperone regulator 3.

BAG3 has several defined domains and binding motifs: from the N-terminal, there is a WW domain, two Ile-Pro-Val (IPV) and two Arg-Ser-Gln-Ser (RSQS) sequence motifs, and a PXXP repeat, which is finally followed by the BAG domain (Figure 6). These allow BAG3 to work as a scaffold that brings together a wide range of interacting proteins for a plethora of cellular functions [16,465].

The WW domain interacts with proline-rich proteins containing the motif [AP]-P-P-[AP]-Y, and in the context of this review, the interaction with PPPY in SYNPO2 (also known as myopodin) is of particular interest [466]. The WW domain also allows BAG3 to bind to YAP/TAZ inhibitor proteins [466,467], thereby affecting the Hippo signaling pathway and also the expression of the actin cross-linking protein filamin. BAG3 also contains a second WW domain annotated in UniProt and clearly fulfilling the domain definition, but this domain has not been studied and is not considered in most reports on BAG3. Thus, the role of this second potential WW domain remains elusive.

The two IPV motifs bind to small heat shock proteins of the HSPB family [159]. The various HSPB proteins have different affinity for BAG3, and HSPB8 has been shown to be the preferred partner [18]. In addition, HSPB1 has been observed to colocalize with filamin C (FLNC) and BAG3 in sarcomere lesions in mice [468]. Functional interactions between HSPB6 and HSPB8 with BAG3 have been studied using a polyQ-HTT client protein, and the presence of IPV motifs is essential for the polyQ-HTT degradation activity of HSPB6 and HSPB8 [159]. However, all HSPB proteins are able to bind to BAG3, and the removal of both IPV elements by either mutation or deletion is required to abolish binding [16].

The two RSQS motifs bind 14-3-3 proteins [469,470]. This allows 14-3-3 proteins to connect BAG3 with the intermediate chain of the dynein complex, facilitating HDAC6-independent aggresome formation [469]. Mutational analysis showed that the mutation of p.S136 reduces binding, whereas p.S173 mutations removed binding to the 14-3-3γ protein [469].

The PXXP repeat region allows BAG3 to interact with SH3-domain-containing proteins [471]. The microtubule motor protein complex dynein interacts directly with the PXXP domain of BAG3 [470,472], allowing the transport of BAG3-bound client along microtubules to the aggresome for degradation. Deletion of the PXXP domain disrupts this function [470] and abolishes the ability of BAG3 and HSPB8 to prevent polyQ-HTT aggregation [15]. Similarly, the depletion of BAG3, HSPB8, or HDAC6 prevents aggresome formation under prolonged proteasomal inhibition, and depleted cells show dispersed ubiquitin-positive cytoplasmic puncta [408].

The BAG domain, as already mentioned, is mostly responsible for the interaction of BAG3 with HSPA, and for the NEF activity [158]. The domain also interacts with e.g., the anti-apoptotic protein BCL2 [460] and the transcription factor HSF1 [473].

BAG3 has several reported or predicted sites for post-translational modifications. Lys445 in the BAG domain is identified as a SUMOylation site and cross-links with small ubiquitin-like modifiers SUMO1 [474] or SUMO2 [475]. BAG3 can also be ubiquitinated by STUB1 [421]. Several phosphorylation sites have been identified. Of particular importance are the phosphorylation sites at Ser136 and Ser173, which directly affect binding to the 14-3-3γ protein [469]. In addition, two methylation sites are predicted from similarity with mouse Bag3. However, the precise functional role and regulation of these sites are not known. A caspase cleavage site is located at Asp347; cleavage at this site causes the loss of the anti-apoptotic function of BAG3 [476].

#### 4.1.1. Regulation of BAG3 Expression 

BAG3 is the only stress-inducible BAG protein [477]. Transcription factors such as HSF1 bind to BAG3 upon stress and translocate to the nucleus, inducing the expression of a range of heat shock proteins, including BAG3 itself [461,473,478]. BAG3 expression is also modulated by the nuclear factor κB (NF-κB) during stress recovery [479].

Proteasome inhibition induces autophagy [470,480,481], and increased expression of the *BAG3* gene has been observed with a reduced level of BAG3 protein consistent with co-degradation of BAG3 during autophagy [421,482]. On the other hand, lysosomal inhibition does not affect *BAG3* expression, but consistent with autophagic turnover, it leads to an increased level of the BAG3 protein [421]. Since *BAG3* expression is upregulated by proteasomal inhibition and leads to the redirection of proteasome-targeted clients to macroautophagy, a mechanism been called BiPASS (“BAG-instructed Proteasomal to Autophagosomal Switch and Sorting) has been suggested [399].

The level of BAG3 is regulated during mitosis by HSPB8, and the HSPB8–BAG3 complex is important for actin handling during cytokinesis [483].

BAG3 shows higher expression in several aggressive tumor types and is upregulated under oxidative as well as proteotoxic stress [484]. In cancers, high expression of BAG3 has been linked to resistance to chemotherapy and knockdown with increased sensitivity [485].

#### 4.1.2. Regulation of Expression by BAG3

BAG3 modulates the mTORC signaling pathway by sequestering the mTORC1 inhibitors TSC1 and TSC2, allowing for the simultaneous local activation of autophagy and protein synthesis during mechanical strain and exercise [486]. BAG3 binds TSC1 and TSC2 via the WW domain [486], which is the same domain that also binds SYNPO2 [466]. Hence, the WW domain is of critical importance for the regulation of transcription as well as CASA, and interestingly, no disease mutations have been identified in this region. The depletion of SYNPO2 releases BAG3 from its autophagic roles and upregulates YAP/TAZ-mediated transcription [466]. It was recently reported that the BAG3–HSPA complex is critical in the LATS1/2-mediated phosphorylation of YAP, which is indicative of Hippo pathway activation [487].

It has been shown that some of the beneficial effects observed by BAG3–HSPB8 overexpression is via the eIF2α signaling pathway, leading to induced autophagy and inhibited protein synthesis [422].

#### 4.1.3. BAG3 Proteostasis and Transport

Through interactions with HSPA proteins and SQSTM1, BAG3 is directly linked to the proteostasis machinery. The depletion of BAG3 in cells elevates the basal level of polyubiquitinated proteins and also redirect client proteins to proteasomes for degradation [488].

The aggresome is a collection of accumulated proteins located around the microtubule organizing center (MTOC). BAG3 has been shown to promote the sequestration of ubiquitinated client proteins to the aggresomes [470]. These proteins are retro-transported by dynein complexes along microtubules and collected for later autophagosomal degradation. However, also non-ubiquitinated proteins are found in the aggresome, and BAG3 has been suggested to be involved in the ubiquitin-independent process as well [470]. HDAC6 is important for binding poly-ubiquitinated proteins to the dynein motors, and HDAC6-deficient cells fail to form aggresomes and remove misfolded cytoplasmic proteins [489]. Both the BAG3-mediated as well as the HDAC6-mediated client transport to aggresomes depend on dynein. HSPB8-bound ubiquitinated client proteins are upon binding between HSPB8 and BAG3 targeted to the aggresomes, but interaction between HSPB8 and BAG3 is not strictly required for aggresome formation [408]. The results presented by Guilbert et al. showed that BAG3 interacts with SQSTM1 independently of HSPB8 SQSTM1 [408], but the interaction between HSPB8 and BAG3 is required for the efficient coupling of SQSTM1 bodies (p62 bodies) for transport to aggresomes under proteasomal stress [408]. Proteasomal inhibition increased the level of phosphorylated SQSTM1, which is important for the control of ubiquitinated inclusion formation, and this was partially reduced by the silencing of either HSPB8 or BAG3, and they concluded that HSPB8 and BAG3 facilitates the stress-induced sequestering activity of SQSTM1 [408].

In cell studies, the chemical inhibition of dynein function was shown to counteract the autophagy induction caused by trehalose, indicating that dynein repression causes autophagy reduction [417]. Inhibition of retrograde transport was, as expected, found to reduce the aggregation of mutant proteins in the aggresome but also to increase their clearance [417]. The inhibition correlated with a strong induction of BAG1, indicating a switch to the proteasomal degradation of mutant proteins when the preferred autophagosomal pathway and transport to the aggresome was blocked [417]. Studies of mutant SOD1 have shown that the HSPA-bound mutant SOD1 is transported to the aggresomes by BAG3 and dynein, and that a BAG3-derived construct containing only the dynein-binding PxxP-motif and the HSPA-binding BAG domain is sufficient for this function [470].

#### 4.1.4. BAG3 in Autophagy

Chaperone-assisted selective autophagy (CASA) is a selective/targeted degradation pathway guiding chaperone-bound ubiquitinated proteins to the lysosome [421]. It was originally described as a pathway mediated by Starvin, the *Drosophila* BAG3 ortholog; hence, BAG3 is a crucial partner of this pathway, which also contains HSPB8, HSPA8, STUB1, and SQSTM1 [421].

During CASA, client proteins are recognized by BAG3, HSPA8, and HSPB8; then, the client protein is ubiquitinated by the E3 ligase STUB1, followed by sequestration to the aggresome [466,482]. SQSTM1 links the CASA complex to the phagophore membrane following SYNPO2 interaction with BAG3 autophagosome formation around the CASA complex, resulting in the eventual co-degradation of CASA proteins with their clients [466,482].

CASA is important for maintaining the structural integrity of muscle cells [421,490] and senses mechanical tension through interaction with the client protein filamin [466]. CASA components are induced upon strenuous physical exercise [482,491]; also the electrostimulation of isolated muscle fibers increases BAG3 levels and high-molecular-weight ubiquitin conjugates [421]. These conjugates accumulate in microaccumulations that partially co-stain with LC3, suggesting CASA induction [421].

Trehalose has been recently used in studies for the reduction of aggregation phenotypes in both cells and animals [419,492,493]. Trehalose induces transient lysosomal enlargement, and damaged lysosomal membranes are visible by electron microscopy [494]. Trehalose also induces the expression of BAG3, HSPB8, and SQSTM1 [494]—all key components of the CASA pathway. The induction of SQSTM1 expression was found to be TFEB-dependent, whereas BAG3 and HSPB8 induction were not [494]. In C2C12 cells, the inhibition of autophagy by bafilomycin A reverted the aggregate-reducing function of trehalose, illustrating that the effect of trehalose is mediated by autophagy [419].

The kinase STK38 was recently shown to inhibit CASA activity by binding to BAG3, causing a loss of interactions with SYNPO2 and HSPB8 but without affecting binding to HSPA8 [495]. This inhibitory role is dependent on the phosphorylation of Thr444 in STK38 [495].

#### 4.1.5. Stress Granules and Defective Ribosomal Products

Stress granules (SGs) are stress-induced ribonucleoprotein complexes that sequester mRNA temporarily for translation at a later time. The dynamics of stress granules are highly important for proper cellular function, and there are several reports of disease caused by mutations affecting SG dynamics [496,497,498]. Whereas normal SGs dissolve after stress, releasing their mRNA and protein content for other functions, aberrant SGs that have lost their dynamic behavior need to be removed by autophagy [407]. The accumulation of defective ribosomal products (DRIPs)—translated proteins unable to reach a native state for any reason—has been shown to promote an aberrant behavior of SGs [407].

The HSPB8–BAG3–HSPA complexes are important for proper SG function and restore proteostasis by promoting degradation of DRIPs [407]. Ganassi and coworkers induced SG formation by arsenite, causing oxidative stress, or MG132, causing proteotoxic stress by proteasomal inhibition [407]. HSPB8 was found in all TIA1-positive SGs, whereas BAG3 was only rarely present [407]. However, BAG3 was found in DRIP-containing stress-induced SGs that were largely devoid of HSPB8 [407]. The proposed mechanism involved HSPB8 acting as a chaperone inside SGs and preventing DRIP aggregation, allowing for later degradation by the BAG3–HSPA machinery [407].

Even if the disassembly of SGs and targeted degradation of aberrant proteins are preferred, some SGs, especially if they contain DRIPs, are transported by BAG3 and SQSTM1 to aggresomes for degradation [407]. This illustrates that at least under certain conditions, there is a clear link between proteins containing prion-like domains and the autophagic machinery.

The effect of BAG3 on SGs and its role in neurodegenerative disorders was recently reviewed by Duggan et al. [499].

#### 4.1.6. BAG3/BAG1 Ratio and Aging

Whereas BAG3 targets clients to the autophagosome and prevents proteasomal degradation of HSPA-bound clients, BAG1 directs them to the proteasome [12]. Both BAG1 and BAG3 interact with many of the same chaperone partners, leading to competition between the two BAG proteins for binding sites. Therefore, the levels of functional BAG1 and BAG3 in the cells are important for the balance between proteasomal and lysosomal turnover. The BAG3/BAG1 ratio changes during aging—with samples from older individuals showing increased BAG3 and decreased BAG1 levels—causing a shift from proteasomal toward autophagic degradation [399,488]. A similar shift from BAG1 to BAG3 expression and from proteasomal to autophagic degradation has also been observed in several cell types under stress conditions, e.g., in cells adapted to peroxide-induced oxidative stress [399,484].

The accumulation of oxidized proteins is a feature of cellular aging. These proteins are also more likely to form cross-links, thereby largely preventing proteasomal degradation and leading to a greater importance of the autophagic pathways.

The increase in BAG3 expression is mirrored by an increased expression of SQSTM1 [488]. However, this increase in SQSTM1 level is not induced by changes in the BAG3 level, as knock-down of BAG3 in old cells induces a stress response including the increased expression of SQSTM1 [488]. In brain, the increased BAG3/BAG1 ratio is followed by increased cathepsin activity as well as LC3-II levels [488].

Maintaining a proper balance between proteasomal (BAG1) and autophagic (BAG3) degradation in relation to soluble and insoluble client proteins could be of greater importance than the absolute cellular level of (co-)chaperones [488].

#### 4.1.7. Other Functions of BAG3 in Muscle Cells

BAG3 has also other roles in muscle cells. It has been shown to regulate myofibril stability through HSPA8, causing a tight interaction between F-actin and CAPZ [500]. BAG3 is the driver for HSPA8 localization to the juxtamembrane region, where CapZ and F-actin are enriched, and this function is dependent on the BAG domain of BAG3 [500]. The absence of BAG3 causes the misslocalization and proteasomal degradation of CapZ proteins [500].

BAG3 is also, at least in cardiomyocytes, involved in contraction and affects calcium signaling through interactions with calcium channels [501,502]. Whether BAG3 is similarly involved in calcium signaling in skeletal muscle is not known.

### 4.2. BAG3 in Neuromuscular Disease

The first report of disease-causing mutations in BAG3 appeared more than 10 years ago when the same p.P209L mutation was identified as a *de novo* change in three unrelated patients in a cohort of undiagnosed MFM patients [464]. Since then, many mutations in BAG3 have been published (Table 9). All mutations reported so far have been in heterozygous state, and the vast majority is found in dilated cardiomyopathy cases. However, mutations affecting Pro209 and Pro470 cause myopathy [106,464,503,504,505,506,507,508,509,510,511,512,513,514]. The cardiomyopathy mutations and the role of BAG3 in the heart have been recently reviewed by others [485,515,516]. We will focus here on the mutations causing neuromuscular disease.

Most reported p.P209L patients show an early disease onset (first or second decade), rapid progression, and a severe pathology—including cardiomyopathy often requiring hearth transplantation, as well as respiratory involvement. Of the three patients in the original report, one received a heart transplant at age 13 and one died at age 13 following a respiratory infection [464].

So far, only one published case of a classical BAG3 p.P209L myopathy is described without cardiomyopathy at age 25, which is an age where all other p.P209L patients had either died or had clear cardiomyopathy [509]. However, occasionally, BAG3 p.P209L patients present with non-myopathic symptoms, and several axonal neuropathy cases have been reported [507,508,512]. These patients tend to get an initial diagnosis of CMT and often present with rigid-spine syndrome and sensory-motor neuropathy. Upon deeper clinical evaluation, they do show myopathic features and clear ultrastructural changes such as Z-disc streaming and filamentous accumulations, and some are reported without signs of cardiomyopathy [508,512].

The severity of classical BAG3 myopathy is illustrated by the fact that the p.P209L mutation has been found to be *de novo* in all but two reported cases. In one family, the allele that was found mutated in two affected brothers was inherited from the non-mutation carrying father, indicating mosaicism [505]. In another family, the father and one sister of the index patient had a similar, but much milder, phenotype, indicating another case of potential mosaicism [507]. However, in the latter report, the relatives were not available for clinical or genetic evaluation [507].

The p.P209Q (c.626C>A) mutation has been reported in one patient with relatively mild myopathy and no cardiomyopathy at age 43 [503]. On the cellular level, typical MFM findings were seen: Z-disc streaming, granulofilamentous accumulations, desmin-positive protein deposits, and vacuoles. The patient did also have axonal sensorimotor polyneuropathy, which was in line with several of the p.P209L cases described above. Genetic analysis of the parents showed that the c.626C>A mutation was *de novo* [503].

The p.P209S (c.625C>T) mutation has been identified in one individual [504], two large families with peripheral neuropathy [519], and one family with axonal CMT [520]. No involvement of heart or skeletal muscle was observed in the two families, but the pedigrees included persons who died of cardiac disease and cardiac problems could not be ruled out [519]. The single individual was reported with adolescence-onset polyneuropathy, with signs of atrophy of the lower legs [504].

The p.Pro407Ser mutation, located in the BAG domain, was recently reported in two unrelated cases as a cause of MFM [106]. The mutation was proven *de novo* in one case, but as only one of the relatives was available for the other patient, the inheritability in the latter case is unknown. In both cases, the onset was around age 30, and the biopsies showed typical MFM findings such as sarcoplasmic inclusions and rimmed vacuoles. Both patients had neurogenic changes, but no heart-related pathology was reported [106].

#### 4.2.1. BAG3 Animal Models

##### BAG3-Deficient Models

In zebrafish, BAG3 knock-down by morpholino injection has been found to cause cardiac phenotypes [517] and contraction-dependent myofibrillar disintegration [526]. On the other hand, zebrafish model haploinsufficient for BAG3 showed elevated levels of ubiquitinated proteins, indicating impaired autophagic flux, and developed dilated cardiomyopathy (DCM) [527]. Transcriptome analysis of heart samples identified the upregulation of the mTOR signaling pathway, and crossing the mutant fish with an mTOR-haploinsufficient mutant restored levels of ubiquitinated proteins, restored life span, and reduced the cardiac phenotype, demonstrating that modulation of the mTOR pathway can be beneficial for BAG3 haploinsufficiency [527].

In mice, homozygous disruption of the *Bag3* gene showed that BAG3 is not required for the normal development of heart and skeletal muscle [108]. At birth, the BAG3-deficient mice are normal, but they soon start to develop clear signs of myopathy starting with disturbed Z-discs and sarcomeric disarray already at day 4 [108]. This progresses to also include apoptotic features after 2 weeks, ultimately leading to death by day 25 [108]. The observed pathologies were limited to myofibers, and the severity of muscle damage correlated with muscle use, indicating that BAG3 is especially important for maintenance of the muscle integrity in actively used muscles [108]. No evidence for muscle necrosis and only few regenerating fibers were identified in the BAG3-deficient mice, and the creatine kinase level was within normal range consistent with a lack of sarcolemmal damage [108]. In addition, no evidence of neuropathy was observed in this mouse model [108].

In another BAG3-deficient mouse model with an introduced deletion of exon 4 in *Bag3*, there was little evidence of apoptosis, but instead a dramatic metabolic shift was observed [528]. These BAG3-deficient mice were hypoglycemic and had lipid accumulations in the liver indicating nutrient insufficiency, but no muscle pathology of note was observed [528]. The BAG3-deficient mice had reduced growth, and all homozygous deletion mice died before age 3 weeks [528]. Why the two mouse models were phenotypically different is not known, but Youn and coworkers suggested that differences in the gene-targeting method could be the reason [528].

The cardiac phenotype observed in patients as well as the mouse model developed by Homma et al. [108] was recapitulated in a cardiac-specific homozygous knock-out model [406]. These mice suffer from cardiomyopathy, but with normal BAG3 expression in skeletal muscle survives until around 10 months [406]. Another, heterozygous cardiac-specific haploinsufficiency model recapitulated the DCM phenotype seen in patients with BAG3 truncations or deletions [472].

##### BAG3 Mutation Models

The BAG3 myopathy mutation p.P209L was studied extensively in zebrafish by muscle-specific overexpression of GFP-tagged human BAG3 under the *acta1* promoter [526]. Both mutant and wild-type BAG3 were localized to the sarcomeric Z-disc, but the mutant fish also showed small, granular accumulations from 32 hours post fertilization [526]. Consistent with the Pro to Leu mutation being predicted to reduce thermostability, keeping the fish at 37 °C compared to the normal 28 °C increased the size of the accumulations [526]. Further studies of the accumulations revealed both wild-type BAG3 as well as FLNC [526]. The presence of wild-type BAG3 suggests that part of the pathologies observed might come from haploinsufficiency [526]. In fluorescence recovery after photobleaching (FRAP) analysis, the recovery of mutant BAG3 was reduced, but it was not significantly different from the wild-type [526]. Autophagy inhibition by chloroquine resulted in a significant increase in the fraction of fibers with BAG3 accumulations, whereas induction by rapamycin caused a significant decrease, showing that the observed pathologies in the fish are not a result of autophagic dysfunction [526].

The recently reported mouse model with a p.P215L mutation in Bag3 (equivalent to the p.P209L in human BAG3) showed neither a cardiac phenotype at 16 months of age nor any change in other measured parameters such as life span, weight, or growth even in homozygous state [529]. This lack of phenotypes lead Fang et al. to suggest that the pathologies caused by p.P209L in humans are species specific [529].

#### 4.2.2. Pathomechanisms

It is intriguing that the mutations in BAG3 affecting skeletal muscle are constrained to two Pro residues at positions 209 and 470. Pro209 is in the second IPV-motif, which is important for interactions with the HSPB-family proteins; therefore, mutations in this location might directly affect the binding of HSPBs. In CoIP experiments, the p.P209L mutation was indeed found to reduce binding efficiency, but binding was observed, and hence a simple loss of HSPB-binding was suggested to not be the direct cause of disease [106].

Most morphological studies on BAG3 myopathy have been performed on skeletal muscle biopsies. However, a recent analysis of an explanted heart from an 8-year-old patient with a p.P209L mutation indicated altered autophagy as the main reason for cardiomyopathy and for the quick progression seen in this patient [514]. Western blotting showed substantially increased levels of ubiquitinated proteins, SQSTM1 and LC3-I, but not LC3-II, suggesting insufficient autophagic induction [514].

The p.P209L mutation has been shown to impair the differentiation of the murine skeletal muscle cell line C2C12, but it does not affect the H8c2 cardiomyocyte cell line nor neonatal rat cardiomyocytes (NRCs) [521]. However, there is no indication that myogenesis *per se* is affected by BAG3 mutations. In NRCs, the p.P209L mutant protein is located to the Z-disc in the same way as wild-type, but this is in stark contrast to cells expressing cardiomyopathy mutations that have been found to localize in the nucleus [521]. The cardiomyopathy mutations also affected sensitivity to apoptosis and the Z-disc assembly, whereas the myopathy mutation p.P209L did not [521].

Mutant BAG3 sequesters the wild-type BAG3 protein to aggregates, causing a further loss of functional BAG3 [526]. In zebrafish, the resulting protein accumulations could be reduced by induction of autophagy [526]. Ruparelia and coworkers suggested that even if the myofibrillar disintegration is the direct cause of muscle weakness, the aggregation of mutant BAG3 and sequestration of large parts of the proteostasis machinery is the root cause [526].

Samples from HeLa cells expressing BAG3 p.P209L showed higher levels of BAG3, SQSTM1, and HSPB8 in the insoluble fractions compared to wild-type expressing cells, indicating that the mutant protein sequesters significant parts of the CASA complex and also deregulates the phosphorylation of SQSTM1 [408]. The deregulation of phosphorylation of Ser349 in SQSTM1 cause the mutant BAG3 protein to aberrantly sequester KEAP1 and thereby impair the stress-sensing mechanism that regulates SQSTM1-KEAP1-Nrf2 signaling [408].

The sequestering of chaperone protein partners by BAG3 p.P209L mutant was shortly after confirmed in HEK293 cells by Meister-Broekema and coworkers, who in addition to BAG3 and HSPB8 found DNAJB6b, HSPA1A, and HSPA8 in the insoluble fraction [106]. They also found the p.P209Q, p.P209S, and p.P470S mutations to sequester the same proteins, thereby extending the finding to all myopathy-causing mutations in BAG3 [106]. The p.P470S mutation was found to reduce the binding of BAG3 to HSPA, but it did not abolish it [106]. An engineered p.R480A mutation in the BAG domain of BAG3 did abolish BAG3-HSPA binding [106]. The aggregation phenotype in cell culture was found to be driven by interaction between BAG3 and HSPA; hence, the sequestration could be prevented genetically by introducing the p.R480A change to mutant BAG3 [106] or chemically by blocking the BAG3–HSPA interaction with JG98 [106] or YM1 [487].

The DCM-causing mutation p.E455K is located in the BAG domain and has in cardiomyocytes been found to abolish the binding of BAG3 to the NBD of HSPA1A/B and reduce the binding of BAG3 to HSPA8 [406].

In cell experiments with cardiomyocytes, no Z-disc disturbances are observed with the p.P209L mutation, but these disturbances are easily observed in patient muscle biopsies and in the zebrafish model of Ruparelia et al. [526]. A likely explanation for this is the lack of mechanical stress in cultured cells, which is present in all muscles and probably is needed to induce structural disturbance.

### 4.3. New Results on the Effects of BAG3 p.P209L on DNAJB6

**Figure 7:**
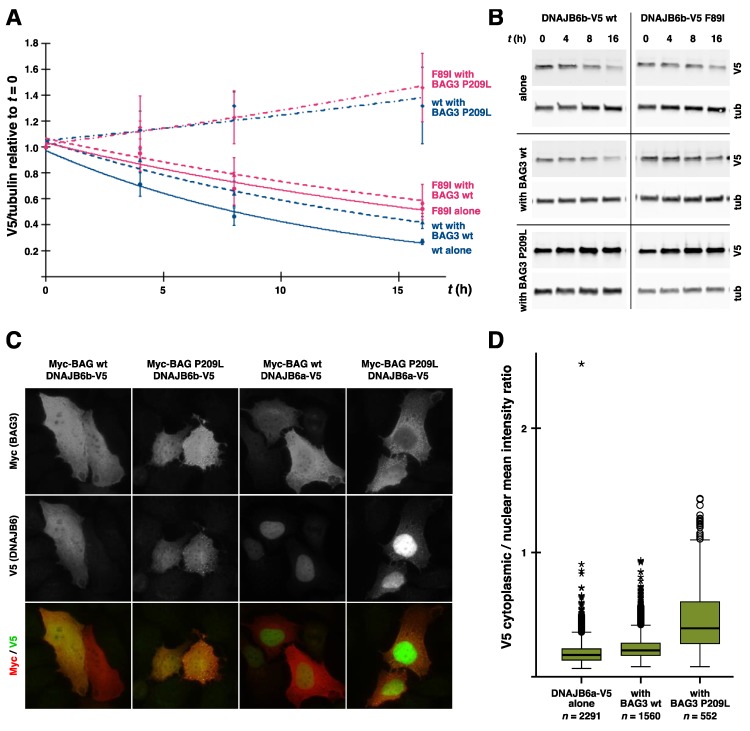
Effect of BAG3 p.P209L on DNAJB6. (**A**) Coexpression with BAG3 p.P209L similarly blocks the turnover of wild-type and p.F89I mutant DNAJB6b. Graph shows mean ± S.D. from 4–5 experiments performed in triplicate, with exponential trendlines fitted to the data points. (**B**) Representative Western blots showing DNAJB6b-V5 wt and p.F89I (V5) and tubulin (tub) levels at different time points. (**C**) Both DNAJB6a and DNAJB6b are recruited to cytoplasmic aggregates formed by BAG3 p.P209L in HeLa cells. (**D**) BAG3 p.P209L causes increased cytoplasmic localization of DNAJB6a in HeLa cells. Box plots show the cytoplasmic/nuclear V5 mean intensity ratio in *n* = 552–2291 cells per group.

As described above, LGMD-causing mutations in DNAJB6 are associated with slower DNAJB6 turnover [14,49]. On the other hand, wild-type but not p.P209L mutant BAG3 was suggested by zebrafish studies to augment the toxicity of mutant DNAJB6 [14]. To determine if this effect of BAG3 could be through the modulation of DNAJB6 turnover, we coexpressed DNAJB6b and BAG3 constructs in an inducible cell system and followed DNAJB6b turnover after construct deinduction. In this system, DNAJB6b p.F89I, as expected, showed slower turnover compared to the wild-type construct, but this was not significantly affected by coexpression of wild-type BAG3 (Figure 7). In contrast, BAG3 p.P209L caused a dramatic block to the turnover of both wild-type and mutant DNAJB6b.

We reasoned that mutant BAG3 might affect DNAJB6b turnover by sequestering it to aggregates. This was supported by microscopic analyses, where DNAJB6b colocalized with BAG3 p.P209L in cytoplasmic puncta (Figure 7). Also the nuclear DNAJB6a isoform was in some cells recruited to BAG3 aggregates, and this observation was confirmed by image analysis (Figure 7). In parallel to our experiments, similar results were obtained by Meister-Broekema et al., who also observed the sequestration of DNAJB6b to insoluble BAG3 p.P209L aggregates [106]. While their findings did not indicate the recruitment of DNAJB6a to the aggregates, our data suggest that also this isoform may in some conditions be affected by BAG3 p.P209L, with potential downstream consequences on DNAJB6a-mediated functions such as cardiac protection against ER stress [41].

## 5. Conclusions

As evident from the functional work discussed here, mutations in chaperones and cochaperones can cause neuromuscular diseases through a variety of mechanisms. Most mutations appear to affect multiple parallel pathogenetic pathways, which most often involve toxic gain-of-function of the mutated protein. Indeed, of the diseases discussed here, only DNAJB2-related neuropathies seem to depend on pure loss of function. However, in many cases, a loss of protective chaperone function is likely to contribute to the disease pathomechanisms. As a result of the interconnections in the PQC network, mutations in one component will inevitably affect the others, potentially leading to shared pathomechanisms. This is well illustrated by the downstream effects of BAG3 mutations on DNAJB6 and a number of other PQC proteins.

The chaperone-associated diseases are a piece of a larger puzzle of protein conformational disorders, which can either result from mutations predisposing the mutated proteins to aggregation or from defective quality control. Here, of growing interest are diseases associated with aberrant phase separation and the aggregation of prion-like domains, which are present in many SG components and other RNA-binding proteins. Mutations interfering with SG dynamics are associated with myopathy, as well as diseases of the multisystem proteinopathy (MSP) spectrum, which are characterized by protein aggregation pathology variably affecting different tissues [496,497,530]. The mechanistic connections of chaperonopathies to these diseases are highlighted by the accumulation of PrD-containing proteins in affected tissues and the demonstrated ability of DNAJB6, HSPB8 and BAG3 to regulate PrD aggregation and SG dynamics [59,105,407]. The recently reported digenic distal myopathy caused by a SQSTM1 mutation and a variant in the PrD protein TIA1 [452] further demonstrates how a combined effect of variants in PQC and client proteins together can determine the phenotypic outcome. It is likely that similar epistatic effects underlie the differences in expressivity and penetrance also in other cases, especially when same mutations are associated with distinct phenotypes.

## 6. Materials and Methods 

### 6.1. Filter Trap Assay

Filter trap assay was performed essentially as described [14].

### 6.2. DNAJB6 Turnover Assay

For studying the effect of BAG3 on DNAJB6 turnover, T-Rex 293 cells (Life Technologies) were cotransfected with inducible pcDNA5/TO-DNAJB6b-V5 (wt or p.F89I) constructs [14] together with constitutively expressed BAG3 constructs (wt or p.P209L) or an empty plasmid (pHTC). DNAJB6 expression was induced with tetracyclin (1 µg/ml) and after 16 h of expression, de-induced by tetracyclin washout. Cells were harvested 4 h (set as t=0), 8 h, 12 h, and 20 h after Tet washout. Remaining DNAJB6-V5 was quantified from total cell extracts by western blotting and normalized to tubulin.

### 6.3. Microscopy and Image Analysis

HeLa cells cultured on coverslips were cotransfected with wild-type pCDNA5/TO-DNAJB6a-V5 or DNAJB6b-V5 together with pCDNA5/TO-Myc-BAG3 (wt or p.P209L) or the empty pCDNA5/TO vector. For the analysis of DNAJB6 and BAG3 (co)localization, PFA-fixed cells were stained with antibodies against the V5 and Myc tags and imaged with widefield fluorescence microscopy using a 63× oil immersion objective. For quantitative analysis of DNAJB6a-V5 localization, the cells were stained with anti-V5 and Hoechst and imaged using a 10× objective. A home-written ImageJ macro was used for automated quantification of nuclear and cytosolic V5 fluorescence intensity from cells showing moderate DNAJB6a-V5 expression (based on mean nuclear fluorescence intensity).

## Figures and Tables

**Table 1:** Chaperone and cochaperone genes currently known to underlie neuromuscular disorders.

Gene Symbol	Neuromuscular Disorder(s) (MIM^1^ Number When Available)
*BAG3*	MFM6 (#612954); CMD1HH (#613881); CMT2
*CCT5*	Hereditary sensory neuropathy with spastic paraplegia (#256840)
*CRYAB*	MFM2 (#608810); CMD1II (#615184); Fatal infantile hypertonic myofibrillar myopathy (#613869)
*DNAJB2*	DSMA5 (**#**614881); CMT2
*DNAJB6*	LGMD D1 DNAJB6-related (#603511); Distal myopathy with rimmed vacuoles
*HSPB1*	dHMN2B (#608634); CMT2F (#606595)
*HSPB3*	dHMN2C (#613376); CMT2; (neuro)myopathy
*HSPB8*	dHMN2A (#158590); CMT2L (#608673); Neuromyopathy with rimmed vacuoles
*HSPD1*	Spastic paraplegia 13, autosomal dominant (**#**605280)
*SACS*	Spastic ataxia, Charlevoix–Saguenay type (**#**270550)
*SIL1*	Marinesco–Sjögren syndrome (**#**248800)
*STUB1*	Spinocerebellar ataxia, autosomal recessive 16 (**#**615768)
*TOR1A*	Torsion dystonia, early onset (**#**128100)
*VCP*	Scapuloperoneal muscular dystrophy and dropped head syndrome; Distal myopathy; IBMPFD (# 167320); ALS14 (#613954); CMT2Y (#616687)
*VMA21*	X-linked myopathy with excessive autophagy (XMEA) (**#**310440)

^1^ MIM, Mendelian Inheritance in Man (www.omim.org).

**Table 2:** DNAJB6 mutations causing neuromuscular disease.

Domain	cDNA Change	Protein Change	Phenotype	References
J	c.149C>T	p.A50V	distal	[90]
	c.161A>C	p.E54A	proximo-distal	[90]
G/F	c.265T>A	p.F89I	LGMD	[14,92,93,94]
	c.271T>A	p.F91I	LGMD (severe)	[88,95]
	c.271T>G	p.F91V	mild	[92,96]
	c.271T>C	p.F91L	LGMD (severe)	[95,97]
	c.273C>G			[88,92]
	c.277T>A	p.F93I	LGMD	[98]
	c.277T>C	p.F93L	LGMD	[14,86]
	c.279C>A			[14,92]
	c.279C>G			[14,88,89,92,98,99]
	c.284A>T	p.N95I	LGMD	[89]
	c.287C>G	p.P96R	distal–proximal	[86]
	c.287C>T	p.P96L		[100,101,102]
	c.293_295delATG	p.D98del	distal	[89]
	c.298T>A	p.F100I		[103]
	c.298C>A	p.F100V	distal onset	[88]
	c.346+5G>A	p.G79_F115del	severe, early onset	[88]

Reference sequences: NM_058246.4 (nucleotide), NP_490647.1 (protein).

**Table 3:** Functional consequences of DNAJB6 mutations.

Protein Change	PolyQ Aggregation	PARK p.C289G Aggregation	Sis1 Complementation ^1^	[RNQ+] Propagation ^2^	[PSI+] Propagation/Solubility ^2^	TDP-43 Aggregation	hnRNPA2 p.D290V Aggregation ^3^	Hrb98DE Localization & interaction ^3^	Myotoxicity in Zebrafish	References
**A50V**	†††††					†				[90]
**E54A**	††††					†				[90]
**F89I**	††††		††	††/†	††	†	†	†	†††	[14,59,105]
**F91I**	††									[95]
**F91L**	†††								†††	[95]
**F93L**	†	†	–	–	–	†	†		††(†)	[14,31,59,90,105]
**N95I**	††††									[89]
**P96R**	†††††		†††	†/–	†	†			†	[105], this paper
**P96L**	†††									[102]
**D98del**	††††									[89]
**F100I**	†††††									this paper

Number of † symbols indicates severity of observed defect, – no defect observed, (empty) not reported. Results based on corresponding mutations in: ^1^ DNAJB1, ^2^ chimeric Sis1/DNAJB6 construct, ^3^ *Drosophila* dMRJ.

**Table 4:** DNAJB2 mutations causing neuromuscular disease. CMT2: Charcot–Marie–Tooth disease type 2, dHMN: distal hereditary motor neuropathy.

Mutation ^1^	Phenotype	References
c.14A>G (p.Y5C)	CMT2	[113]
c.229+1G>A (splice)	dHMN	[113]
c.310delC (p.R104Gfs*97)	CMT2	[37]
c.352+1G>A (splice)	dHMN, CMT2, parkinsonism	[112,127,128]
c.619-1G>A (splice)	CMT2	[37]
g.219277938_219281781del^2^	dHMN, parkinsonism	[129]

**^1^** Reference sequences: NM_001039550.**1** (cDNA), NP_001034639.1 (protein), NC_000002.12 (genomic) ^2^ Annotation based on Figure 1 in [129].

**Table 5:** αB-crystallin (*CRYAB*) mutations reported to cause muscle or heart disease, and functional characteristics.

Mutation ^1^	Phenotype ^2^	Inheritance ^3^	Thermal Stability	Aggregation	Hyperphosphorylation	Oligomer Size	Hetero-oligom/HSPB1	Hetero-oligom/HSPB6	HSPB8 Interaction	Chaperone Activity	References
c.3G>A (p.M1?)	infantile MFM	R									[234]
c.60delC (p.S21Afs*24)	infantile MFM	R									[232]
c.325G>C (p.D109H)	MFM + DCM + cat	D									[225]
c.2326A>C (p.D109A)	MFM (+ DCM) + cat	D									[226]
c.326A>G (p.D109G)	AxM + RCM	D		+							[227]
c.343delT (p.S115Pfs*14)	infantile MFM	R		+							[233,274]
c.358A>G (p.R120G)	MFM + HCM + cat	D	–	++	+	+	+	– – –	+	–	[167,222,250,253,257,267,272,294]
c.451C>T (p.Q151*)	MFM	D	–	+	+	– – –		–	–	+/–	[223,258,259,272]
c.460G>A (p.G154S)	DM/DCM	D	–			=	=	=		+/–	[224,229,261]
c.464_465delCT (p.P155Rfs*9)	MFM	D	–	++	+	– –		–	–	+/–	[223,258,259,272]
c.470G>A (p.R157H)	DCM	D	–	+		–	=	–		+/=	[228,261]
c.527A>G (p.*176Wext*19)	DCM + cat	D									[295]

^1^ Reference sequences: NM_001885.3 (cDNA), NP_001876.1 (protein) ^2^ MFM/AxM/DM, myofibrillar/axial/distal myopathy; DCM/RCM/HCM, dilated/restrictive/hypertrophic cardiomyopathy; cat, cataract ^3^ D, dominant; R, recessive.

**Table 6:** Functional consequences of HSPB1 mutations. NTD: N-terminal domains, CTD: C-terminal domains.

Domain	Mutation ^1^	Inheritance ^2^	Oligomer Size	Oligomer Stability	Oligomer Sensitivity to Phosphorylation	Dimerization	Chaperone Function	Client Binding	Thermal Stability	Aggregation	Cytotoxicity	Cell Stress Tolerance	Effects on Neurofilaments	Effects on Microtubules	Susceptibility to Proteasomal Degradation Mutant HSPB1	References
NTD	P7S	D				=				–			+			[337]
	G34R	D	+	+	–		–									[336,350]
	P39L	D	+	+	–		±				+					[334,350,360]
	E41K	D	+	+	–		–									[336,350]
	G53D	R				=				–			+			[337]
	L58Afs*105	D											=		+	[337,340]
	A61Rfs*100	D											=		+	[337]
	G84R	D	+	–	+		–						(+)			[331,334,348]
	S86L	R														[342]
ACD	L99M	R	+	–	+		–						(+)			[331,334,348]
	R127W	D	+	–	+	–	+	+	–			+		+		[170,333,347,358]
	R127L	D										–				[365]
	Q128R	D				=				–			+			[337]
	D129E	D														[345]
	S135F	D	+	–	+	–	+	+	=		+	+	+	+		[170,333,347,356,358,360]
	R136W	D	+	–		–	+	+	=			=		+		[170,333,347,358]
	R140G	SD	++/–	–		≈	–		–	+	+		(+)			[210,331,334,339,346,360]
	K141Q	D	(+)	–			–		–				(+)			[331,346,368]
	T151I	D				=	=	=				=		=		[170,333,358]
	T164A	D		–			=									[349,369]
	M169Cfs*2	D				=						–			–	[365]
CTD	T180I	D					≈									[335,349]
	P182S	D	+	+		=	–			+			(+)			[331,349,370]
	P182L	D		+		=	=			+		–	+	=		[170,333,351,358]
	S187L	D				=				+			–			[337]
	R188W	D		–			–	=								[336,349]
	A204Gfs*6	D				+	–									[343]

The table shows a selected subset of HSPB1 mutations for which functional information is available. ^1^ Reference sequence NP_001531.1 ^2^ D, dominant; R, recessive; SD, semidominant.

**Table 7:** HSPB3 mutations causing neuromuscular disease.

Mutation ^1^	Phenotype	Effects	Ref
c.21 G>T (p.R7S)	dHMN	slightly altered oligomerization	[378,392]
p.L34Ffs*50 (p.A33Afs*50 in [389])	myopathy	unstable protein, loss of HSPB2 regulation	[389]
p.R116P	myopathy with axonal neuropathy	aggregation, loss of HSPB2 interaction and regulation	[389]
c.352T>C (p.Y118H)	CMT2	not determined, likely loss of HSPB2 interaction and regulation	[393]

^1^ Reference sequences: NM_006308.3 (cDNA), NP_006299.1 (protein).

**Table 8:** HSPB8 mutations causing neuromuscular disease.

Mutation ^1^	Phenotype ^2^	Self-Interaction	HSPB1 Interaction	CRYAB Interaction	BAG3 Interaction	In Vitro Chaperone Act.	In Vivo Chaperone Act.	Aggregation	Cytotoxicity	References
P90L	NP				=			(–)	(+)	[337]
N138T	NP				=			(–)	(+)	[337]
K141E	NP/NMP	+	+	+	(–)	+/–	–	+	++	[20,397,411,433,435,438]
K141M	NP				+			(–)	(+)	[337]
K141T	NP									[437]
K141N	NP	++	++	+	+/–		–	+/(–)	+	[20,337,396,397,411,433,435,448]
Q170Gfs*45	MP									[439]
P173Sfs*43	NMP									[438]

^1^ Reference sequence NP_055180.1; ^2^ NP, neuropathy; MP, myopathy; NMP, neuromyopathy.

**Table 9:** BAG3 mutations reported to cause neuromuscular or cardiac disease.

Mutation ^1^	Phenotype ^2^	References
c.211C>T (p.R71W)	DCM	[517]
c.268C>T (p.R90*)	DCM	[517]
c.326A>G (p.H109R)	DCM	[517]
c.367C>T (p.R123*)	DCM	[517]
delEx3-4	DCM	[518]
delEx4	DCM	[517]
c.625C>T (p.P209S)	CMT	[519,520]
c.626C>T (p.P209L)	MFM	[464,505,506,507,508,509,510,511,512,513,514]
c.626C>A (p.P209Q)	MFM	[503,504]
c.652C>T (p.R218W)	DCM	[521,522]
c.652delC (p.R218Gfs*89)	DCM	[517]
c.727delC (p.H243Tfs*64)	DCM	[523]
c.752delA (p.Q251Rfs*56)	DCM	[524]
c.784G>A (p.A262T)	DCM	[517]
c.913delC (p.M306*)	DCM	[525]
c.925C>T (p.R309*)	DCM	[524]
c.1055delC (p.Q353Rfs*10)	DCM	[518]
c.1135delG (p.G379Afs*45)	DCM	[518]
c.1153_1160delTCTTCCCC (p.S385Qfs*56)	DCM	[524]
c.1181_1182delGA (p.R395fs*48)	DCM	[524]
c.1353C>A (p.T451*)	DCM	[518]
c.1363G>A (p.E455K)	DCM	[518,524]
c.1385T>C (p.L462P)	DCM	[521]
c. 1402G>A (p.V468M)	DCM	[524]
c.1408C>T (p.P470S)	MFM	[106]
c.1430G>A (p.R477H)	DCM	[517]

^1^ Reference sequences: NM_004281.3 (cDNA), NP_004272.2 (protein) ^2^ DCM, dilated cardiomyopathy; CMT, Charcot–Marie–Tooth; MFM, myofibrillar myopathy.

## Abbreviations

ACD	α-crystallin domain
ALS	amyotrophic lateral sclerosis
AxM	axial myopathy
CASA	chaperone-assisted selective autophagy
CMA	chaperone-mediated autophagy
CMT	Charcot–Marie–Tooth disease
CNS	central nervous system
CTD	C-terminal domain
DCM	dilated cardiomyopathy
dHMN	distal hereditary motor neuropathy
DM	distal myopathy
DRIP	defective ribosomal product
ERAD	endoplasmic-reticulum-associated degradation
FRAP	fluorescence recovery after photobleaching
FTA	filter trap assay
G/F region	glycine/phenylalanine-rich domain
HCM	hypertrophic cardiomyopathy
HPD motif	histidine–proline–aspartate -motif
IF	intermediate filament
IPV	Ile-Pro-Val motif
JD	J domain
JDP	J-domain protein (Hsp40)
KI	knock-in
KO	knockout
LGMD	limb-girdle muscular dystrophy
MFM	myofibrillar myopathy
MSP	multisystem proteinopathy
MT	microtubule
MTOC	microtubule organizing center
N2B-us	N2B unique sequence (in titin)
NEF	nucleotide exchange factor
NF	neurofilament
NRC	neonatal rat cardiomyocytes
NTD	N-terminal domain
polyQ	polyglutamine
PTP	permeability transition pore
RCM	restrictive cardiomyopathy
ROS	reactive oxygen species
SG	stress granule
sHSP	small heat shock protein
SMN	survival of motor neuron
S/T region	serine/threonine-rich region
UIM	ubiquitin interaction motif
UPS	ubiquitin-proteasome system
